# CTAS: a network control theory-based approach to identify key regulatory TFs of AS events during epithelial–mesenchymal transition

**DOI:** 10.1093/bib/bbag042

**Published:** 2026-02-10

**Authors:** Yan Gan, Yangsong He, Pu Zhao, Wai-Ki Ching, Yushan Qiu

**Affiliations:** School of Mathematical Sciences, Shenzhen University, 3688 Nanhai Avenue, Nanshan District, Shenzhen, Guangdong 518061 China; School of Mathematical Sciences, Shenzhen University, 3688 Nanhai Avenue, Nanshan District, Shenzhen, Guangdong 518061 China; College of Life and Health Sciences, Northeastern University, 3-11 Wenhua Road, Heping District, Shenyang, Liaoning 110169, China; Department of Mathematics, The University of Hong Kong, Pokfulam Road, Hong Kong; School of Mathematical Sciences, Shenzhen University, 3688 Nanhai Avenue, Nanshan District, Shenzhen, Guangdong 518061 China

**Keywords:** regulatory network, control theory, pseudotime analysis, epithelial–mesenchymal transition

## Abstract

Alternative splicing (AS) is a key driver of transcriptomic diversity and plays a pivotal role in epithelial–mesenchymal transition (EMT). During EMT, dynamic splicing changes contribute to cell plasticity and metastasis, yet the upstream regulatory logic remains unclear. Although transcription factors (TFs) are thought to influence AS programs, they typically act through RNA-binding proteins (RBPs), forming a hierarchical TF$\rightarrow $RBP$\rightarrow $AS cascade. Current computational strategies struggle to recover such multi-layered regulation from bulk cross-sectional data, limiting our ability to identify TFs that ultimately control EMT-related AS events. To address this gap, we developed CTAS, a network control theory-based approach to identify key regulatory TFs of AS events during EMT. CTAS integrates pseudotime ordering, trend analysis, sparse directed network inference, and control-theoretic screening into a unified framework. In simulations, CTAS reconstructs EMT trajectories with Spearman’s $\rho = 0.99946$ and directed networks with ROC AUC = 89.9%, and remains robust under noise. Applied to a TCGA BRCA cohort, CTAS builds a directed TF$\to $RBP$\to $AS network and identifies HOXA3 (1.00), PRDM8 (0.86), and TWIST2 (0.83) as top TF controllers, alongside significant dynamic shifts in nine AS events detected by Wilcoxon test ($P <.05$). A focused CD44 subnetwork further highlights ZNF521 (0.86) and HIC1 (0.65) as candidate regulators. These findings demonstrate that CTAS transforms cross-sectional data into dynamic regulatory insights and yields experimentally testable TFs that control AS during EMT.

## Introduction

Tumor metastasis refers to the spread of cancer cells from the primary site to distant organs, where they form new lesions [1]. Among the multiple molecular and cellular mechanisms involved, epithelial–mesenchymal transition (EMT) is a key process that enables cancer cells to acquire migratory and invasive capabilities. EMT describes the transition of epithelial cells to a mesenchymal phenotype with reduced epithelial characteristics, and it plays critical roles in embryonic development, tissue repair, fibrosis, and tumor metastasis [2, 3]. While the transcriptional regulatory network of EMT has been extensively studied [4–6], the role of AS in this process remains incompletely understood.

AS generates structurally and functionally diverse mRNA and protein isoforms by selecting different splice sites in precursor mRNA [7–9]. AS is regulated by cis-acting elements and trans-acting splicing factors, primarily RNA-binding proteins (RBPs), which bind to specific RNA sequences and influence splice site recognition [10, 11]. Transcription factors (TFs) can modulate AS indirectly by regulating the transcription or activity of RBPs, thereby acting in concert with RBPs to control splicing outcomes in target genes [12, 13]. Systematically uncovering the dynamic regulatory relationships among AS, RBPs, and TFs during EMT is critical to understanding the molecular mechanisms underlying cancer progression.

Gene regulatory networks (GRNs) describe complex gene–gene interactions and have been widely used to analyze biological regulation. Existing GRN inference approaches, including Boolean networks [14–16], differential equation models [17, 18], Bayesian networks [19, 20], association networks [21], and machine-learning-based methods such as GENIE3 [22], which have advanced our understanding of transcriptional control. However, these approaches mainly characterize pairwise or single-layer regulatory relationships and thus cannot fully capture multi-layer dynamics across TFs, RBPs, and AS events. Moreover, most GRN studies identify hub regulators using network topology that reflects structure but not dynamic influence. In contrast, control-theoretic approaches view regulation as a dynamic system, revealing how perturbations such as gene knockouts can steer a network toward specific states. This perspective complements topological analysis and provides mechanistic insight into network controllability [23, 24].

For directed networks, control theory seeks the minimum set of driver nodes whose external inputs can steer the system toward a desired state. Linear or locally nonlinear control tools based on the maximum-matching set identify the smallest set of input nodes required to achieve controllability [25]. When the precise system equations are unknown, feedback-vertex-set (FVS) control schemes can ensure reliable nonlinear control [26, 27], and the extended FVS model jointly considers source and FVS nodes to reduce control cost [28]. For undirected networks, structural controllability can be assessed using the minimum dominating set [29], and recent nonlinear control frameworks such as NCUA [30] have further expanded its applicability. Together, these methods form the theoretical foundation for analyzing control in biomolecular networks.

Despite these advances, the interaction mechanisms among AS events, their regulatory RBPs, and upstream TFs during cancer-associated EMT remain largely unresolved. While several models have explored AS–RBP relationships, most rely on time-series data to infer dynamic regulation that is difficult to obtain in clinical contexts. In contrast, cross-sectional omics datasets are more accessible but lack explicit temporal information, limiting causal inference, and dynamic interpretation. Existing methods for such data mainly rely on co-expression or association analysis, which cannot uncover regulatory causality or reveal key drivers of phenotypic transitions.

Pseudotemporal ordering provides an alternative by reordering cross-sectional samples according to expression similarity to reconstruct latent trajectories of biological progression. This strategy has been effectively used to capture nonlinear cellular dynamics and infer regulatory changes over time. For example, the latent-temporal progression-based Bayesian method inferred GRNs using gene expression and pathological information [31], while the pseudotime causality-based Bayesian model identified dynamic relationships between AS events and RBPs during breast cancer EMT [32]. However, these approaches still lack the ability to integrate TFs into the regulatory hierarchy or quantify regulatory influence.

Building upon these foundations, we develop CTAS, a network control theory-based framework to identify key TFs regulating AS during EMT. CTAS integrates pseudotime ordering, trend analysis, sparse directed network inference, and control-theoretic screening into a unified framework. Using cross-sectional transcriptomic data, CTAS reconstructs dynamic trajectories, infers TF$\rightarrow $RBP$\rightarrow $AS regulatory cascades, and identifies TFs most capable of steering AS dynamics. Applied to simulated and real datasets [33], CTAS reveals hierarchical control mechanisms underlying EMT and identifies potential master TF regulators validated by biological evidence.

The major contributions of this study are as follows: (i) we present the first comprehensive investigation of the TF–RBP–AS regulatory hierarchy during EMT, constructing a dynamic network that reveals a TF$\rightarrow $RBP$\rightarrow $AS cascade; (ii) we employ pseudotime algorithms to reconstruct temporal trajectories from cross-sectional data, transforming static omics into dynamic insights; and (iii) we introduce a structure-based constrained target control (CTC) framework to identify key TFs regulating EMT-associated AS events, providing new clues for potential therapeutic targets in cancer.

## Materials and methods

### Problem definition and method outline

EMT is a hallmark of tumor invasion and metastasis. Dysregulated AS plays an essential regulatory role in EMT, and its aberrations can disrupt normal cellular function and promote tumor progression. AS events therefore hold potential as biomarkers and therapeutic targets in cancer. RBPs regulate AS by binding to cis-regulatory elements within introns and exons, whereas TFs modulate AS indirectly by regulating the expression or activity of RBPs.

**Figure 1:**
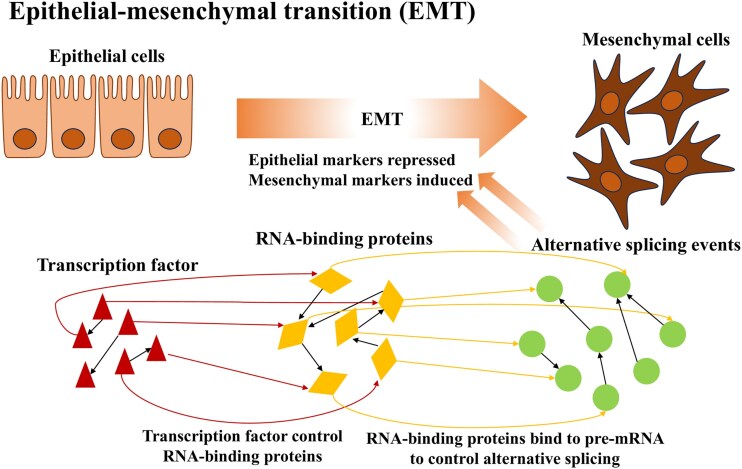
Schematic representation of EMT integrating AS events, RBPs, and TFs.

Figure 1 illustrates the overall EMT process, highlighting the hierarchical regulation among AS events, RBPs, and TFs. Despite their significance, the dynamic regulatory relationships among these molecular components during EMT remain poorly understood. Moreover, obtaining time-series data for EMT is costly, and most cancer transcriptomic datasets lack explicit temporal information.

To address these limitations, we developed CTAS, a control-theory-based framework for constructing and analyzing regulatory networks from cross-sectional data. CTAS reconstructs dynamic expression trajectories via pseudotime analysis, identifies EMT-associated AS events, RBPs, and TFs through trend analysis, builds a dynamic TF–RBP–AS network using ordinary differential equations (ODEs) and mass-action kinetics, and applies CTC to pinpoint key TFs that regulate EMT-related AS events.

### Datasets

We utilized TCGA BRCA Level 3 RNA-SeqV2 gene expression data from the Genomic Data Commons (GDC) Legacy Archive. Details of preprocessing are provided in Text S1. A total of 143 epithelial and 157 mesenchymal samples were selected. Rows with $\geq $100 missing values were removed, and the remaining data were imputed using knnimpute. Rows with zero variance were excluded. The final processed datasets included a $10\,049\times 300$ AS matrix (Table S1), a $1525\times 300$ RBP matrix (Table S2), and a $1500\times 300$ TF matrix (Table S3).

### Overview of the CTAS framework

**Figure 2:**
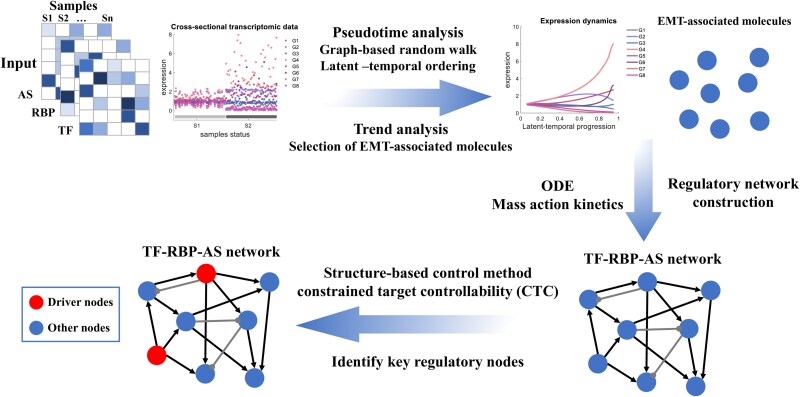
Overview of the CTAS framework.

Figure 2 presents the CTAS workflow that comprises three main modules: (i) pseudotime and trend analysis, (ii) construction of the TF–RBP–AS regulatory network, and (iii) network control analysis to identify key TFs.

In the first module, pseudotime analysis reorders cross-sectional samples to reconstruct latent temporal trajectories. The second module performs trend analysis to detect AS events, RBPs, and TFs exhibiting significant monotonic changes along pseudotime. In the third module, CTAS constructs an ODE-based network model under sparsity assumptions and estimates parameters via Bayesian Lasso regression. Finally, CTC identifies the TFs most capable of steering AS dynamics during EMT.

#### Pseudotime analysis

Pseudotime trajectories were inferred from cross-sectional data using similarity graph-based random walks (Text S2). Epithelial and mesenchymal samples were labeled as 1 and 2, respectively. Each sample was assigned a pseudotime score, enabling reconstruction of a smooth temporal trajectory. The pseudotime order derived from TF expression was subsequently applied to RBPs and AS events, ensuring consistent temporal alignment across molecular layers.

#### Trend analysis

Trend analysis was performed to identify EMT-related AS events, RBPs, and TFs (Text S3). For each molecular feature, we computed the ratio between its linear trend and detrended standard deviation along pseudotime, using the absolute value as its trend score. A higher score indicates greater temporal variation and stronger EMT association. AS events, RBPs, and TFs with the highest trend scores were selected for subsequent modeling.

#### Construction of the regulatory network

The interactions among TFs, RBPs, and AS events were modeled as a dynamic system governed by mass-action kinetics. The system of ODEs is defined as:


(1) \begin{align*} & \frac{\mathrm{d}X_{i}(s)}{\mathrm{d}s}=\sum_{j\neq i}a_{ij}X_{i}(s)X_{j}(s)+\sum_{l=1}^{M}b_{il}X_{i}(s)Y_{l}(s)-d_{i}X_{i}(s), \end{align*}

(2) \begin{align*} & \frac{\mathrm{d}Y_{l}(s)}{\mathrm{d}s}=\sum_{k\neq l}c_{lk}Y_{l}(s)Y_{k}(s)+\sum_{p=1}^{H}e_{lp}Y_{l}(s)Z_{p}(s)-d_{l}^{\prime}Y_{l}(s),\!\!\! \end{align*}

(3) \begin{align*} & \frac{\mathrm{d}Z_{p}(s)}{\mathrm{d}s}=\sum_{p\neq q}g_{pq}Z_{p}(s)Z_{q}(s)-d_{p}^{\prime\prime}Z_{p}(s).\qquad\qquad\qquad\quad \end{align*}

In these equations, $X_{i}(s)$, $Y_{l}(s)$, and $Z_{p}(s)$ denote expression levels of AS events, RBPs, and TFs, respectively, at pseudotime $s$. $a_{ij}$, $b_{il}$, $c_{lk}$, $e_{lp}$, and $g_{pq}$ are dynamic regulatory coefficients, whereas $d_{i}$, $d_{l}^{\prime}$, and $d_{p}^{\prime\prime}$ represent self-degradation rates. Because AS depends on RBPs and RBPs depend on TFs, only the intermediate terms appear in Equations 1,3. The system assumes sparse connectivity consistent with biological networks, and therefore the parameters are estimated using Bayesian Lasso regression, which explicitly enforces sparsity in high-dimensional parameter estimation (Text S4).

#### Control of the regulatory network

Structural control theory was employed to identify key TFs that regulate EMT-associated AS events. The CTC framework determines the smallest set of driver nodes (TFs) required to control a given set of target nodes (AS events) (Text S5). Within CTC [34], the system is represented as:


(4) \begin{align*}& \begin{cases} \dfrac{\mathrm{d}x}{\mathrm{d}t}=Ax+Bu,\\[3pt] y=Cx, \end{cases}\end{align*}

where $x\in \mathbb R^{N}$ and $y\in \mathbb R^{N_{o}}$ denote state variables (nodes) and target outputs. $A\in \mathbb R^{N\times N}$, $B\in \mathbb R^{N\times \phi }$, and $C\in \mathbb R^{N_{o}\times N}$ represent the state-transition, input, and output matrices.

Let $V=\{v_{1},\dots ,v_{N}\}$ denote all nodes, $O$ the target set, and $U$ the constrained control set. CTC identifies the smallest subset $K\subseteq U$ satisfying:


(5) \begin{align*}& \operatorname{rank}\!\bigl([CB, CAB, CA^{2}B,\dots,CA^{N-1}B]\bigr)=N_{o}.\end{align*}

When Equation (5) holds, the system $(A,B,C)$ is said to be constrained-target controllable. Structural controllability is achieved when nonzero entries of $A$ can be freely assigned, ensuring


(6) \begin{align*}& \max\,\Bigl\{\operatorname{rank}\,\bigl([CB, CAB, CA^{2}B,\dots,CA^{N-1}B]\bigr)\Bigr\}=N_{o}.\end{align*}

CTC extends both Kalman and classical target controllability. In this study, EMT-related AS events were defined as target nodes, and TFs were treated as constrained control nodes. A greedy iterative algorithm constructs bipartite graphs to delineate controllable subsystems. The Hopcroft–Karp algorithm then computes maximum matchings and determines control paths from TFs to AS events. The minimal set of driver TFs is identified using a minimum coverage approach and optimized via branch-and-bound linear programming. Finally, Markov-chain sampling produces alternative maximum-matching configurations to evaluate robustness. Figure 2 conceptually summarizes this process, in which different matching configurations yield distinct control paths, allowing robust identification of key TFs across network realizations.

## Results and discussion

### Testing method with a synthetic dataset

**Figure 3:**
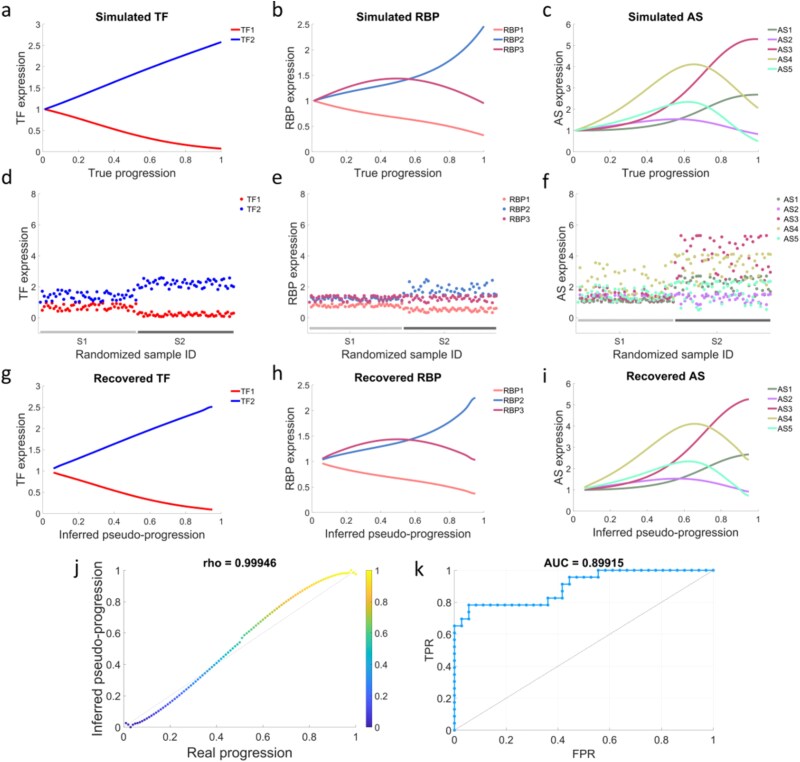
Synthetic dataset demonstration of model capabilities. (a–c) Original TF/RBP/AS expression; (d–f) cross-sectional inputs after random permutation; (g–i) recovered dynamics along inferred pseudotime; (j) correlation between inferred and true pseudotime (Spearman’s $\rho $); (k) ROC–AUC for network inference.

To evaluate performance under cross-sectional sampling (Text S6), we simulated $2$ TFs, $3$ RBPs, and $5$ AS events across $100$ specimens. Figure 3a–c displays the original temporal profiles, and Fig. 3d–f presents randomly permuted cross-sectional inputs used by CTAS. Figure 3j shows that the inferred pseudotime closely matches the ground truth (Spearman’s $\rho =0.99946$). Figure 3g–i demonstrates that recovered TF/RBP/AS dynamics resemble the original trends. Figure 3k reports an AUC of $89.915\%$ for network reconstruction via Bayesian Lasso, indicating accurate recovery of regulatory structure.

To assess robustness, we perturbed TF/RBP/AS values with multiplicative exponential noise (mean $\mu $, $0$%–$10\%$) and varied the coefficient of variation (CV) from $0\%$ to $20\%$. The EMT trajectory was retained and sample IDs were shuffled to emulate cross-sectional acquisition. Figure 3a and b summarizes pseudotime accuracy measured by root mean square error (RMSE) and Spearman’s rank correlation coefficient ($\rho $), and Fig. 3c–f summarizes network metrics including area under the receiver operating characteristic curve (AUC), accuracy, positive predictive value (PPV), and Matthews correlation coefficient (MCC), all indicating stable performance.

We further formalized robustness as follows.

Theorem 1.1. Assume two pseudotime trajectories $s(r)$ and $\tilde{s}(r)$ share root $r\in I=[0,1]$ and define $\|\tilde{s}-s\|_{L^{2}}=(\int _{I}|\tilde{s}-s|^{2}\mathrm{d}r)^{1/2}$. If $(X_{i}(s),Y_{l}(s),Z_{p}(s),a_{ij},b_{il},c_{lk},e_{lp},g_{pq})$ and $(X_{i}(\tilde{s}),Y_{l}(\tilde{s}),Z_{p}(\tilde{s}),\tilde{a}_{ij},\tilde{b}_{il},\tilde{c}_{lk},\tilde{e}_{lp}, \tilde{g}_{pq})$ both satisfy the progression-dependent system

(7) \begin{align*} & \frac{\mathrm{d}X_{i}(s)}{\mathrm{d}s}=\sum_{j\neq i}a_{ij}X_{i}(s)X_{j}(s)+\sum_{l=1}^{M}b_{il}X_{i}(s)Y_{l}(s)-d_{i}X_{i}(s), \end{align*}

 

(8) \begin{align*}\ \ \ & \frac{\mathrm{d}Y_{l}(s)}{\mathrm{d}s}=\sum_{k\neq l}c_{lk}Y_{l}(s)Y_{k}(s)+\sum_{p=1}^{H}e_{lp}Y_{l}(s)Z_{p}(s)-{d^{\prime}}_{l}Y_{l}(s), \end{align*}

 

(9) \begin{align*}\ & \frac{\mathrm{d}Z_{p}(s)}{\mathrm{d}s}=\sum_{p\neq q}g_{pq}Z_{p}(s)Z_{q}(s)-{d^{\prime\prime}}_{p}Z_{p}(s),\qquad\qquad\qquad\ \end{align*}

with $i=1,\dots ,N$, $l=1,\dots ,M$, $p=1,\dots ,H$, and

(10) \begin{align*} & \frac{\mathrm{d}X_{i}(\tilde{s})}{\mathrm{d}\tilde{s}}=\sum_{j\neq i}\tilde{a}_{ij}X_{i}(\tilde{s})X_{j}(\tilde{s})+\sum_{l=1}^{M}\tilde{b}_{il}X_{i}(\tilde{s})Y_{l}(\tilde{s})-\tilde{d}_{i}X_{i}(\tilde{s}),\end{align*}

 

(11) \begin{align*} & \frac{\mathrm{d}Y_{l}(\tilde{s})}{\mathrm{d}\tilde{s}}=\sum_{k\neq l}\tilde{c}_{lk}Y_{l}(\tilde{s})Y_{k}(\tilde{s})+\sum_{p=1}^{H}\tilde{e}_{lp}Y_{l}(\tilde{s})Z_{p}(\tilde{s})-\tilde{d}^{\prime}_{l}Y_{l}(\tilde{s}), \end{align*}

 

(12) \begin{align*} & \frac{\mathrm{d}Z_{p}(\tilde{s})}{\mathrm{d}\tilde{s}}=\sum_{p\neq q}\tilde{g}_{pq}Z_{p}(\tilde{s})Z_{q}(\tilde{s})-\tilde{d}^{\prime\prime}_{p}Z_{p}(\tilde{s}),\qquad\qquad\qquad\quad \end{align*}

with $i=1,\dots ,N$, $l=1,\dots ,M$, $p=1,\dots ,H$, then we have

(13) \begin{align*} & \lim_{\|\tilde{s}-s\|_{L^{2}}\rightarrow0}\left(\sum_{j=1}^{N}(\tilde{a}_{ij}-a_{ij})^{2}+\sum_{l=1}^{M}(\tilde{b}_{il}-b_{il})^{2}\right)=0, \end{align*}

 

(14) \begin{align*} & \lim_{\|\tilde{s}-s\|_{L^{2}}\rightarrow0}\left(\sum_{k=1}^{M}(\tilde{c}_{lk}-c_{lk})^{2}+\sum_{p=1}^{H}(\tilde{e}_{lp}-e_{lp})^{2}\right)=0, \end{align*}

and

(15) \begin{align*}& \lim_{\|\tilde{s}-s\|_{L^{2}}\rightarrow0}\left(\sum_{q=1}^{H}(\tilde{g}_{pq}-g_{pq})^{2}\right)=0.\end{align*}

**Figure 4:**
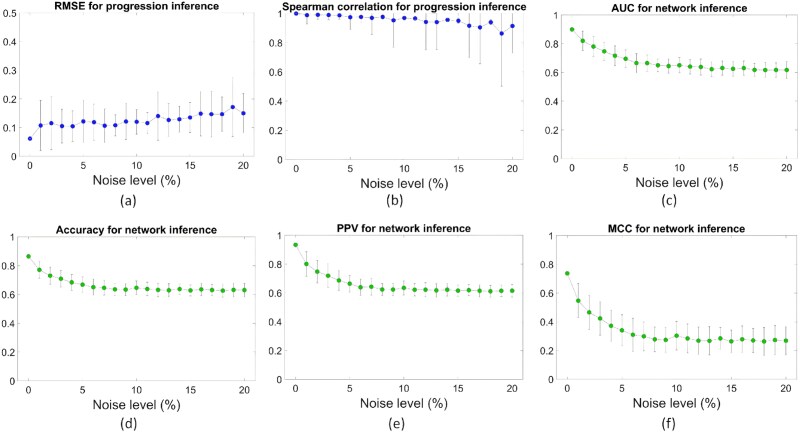
Robustness assessment using synthetic noise. CV varied from $0\%$ to $20\%$. (a and b) RMSE and Spearman’s $\rho $ for pseudotime; (c–f) AUC, accuracy, PPV, and MCC for network reconstruction.

The proof is provided in Text S7. Figures 3 and 4 together show that CTAS accurately recovers pseudotemporal order and regulatory structure and remains stable under substantial variability.

### Construction and analysis of regulatory networks

**Figure 5:**
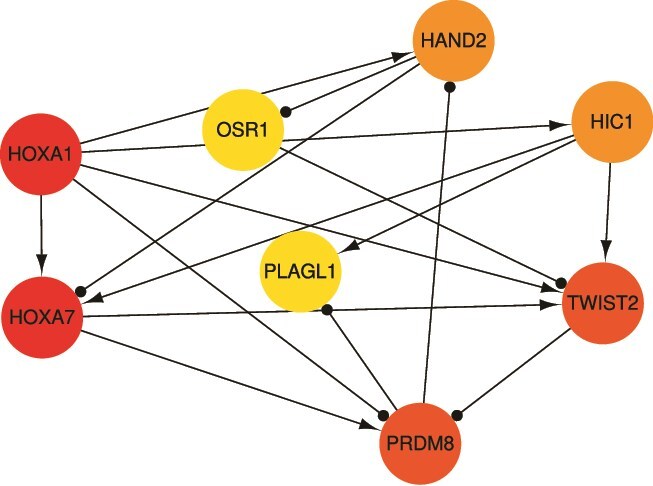
The TF regulatory network.

We applied CTAS to the breast cancer dataset in [33]. Pseudotime ordering placed epithelial samples at the trajectory start and mesenchymal samples at the end (Table S4), whereas pathological stage showed little alignment. Trend analysis then scored TFs/RBPs/AS events, and we selected the top 50 AS events, 10 RBPs, and 10 TFs for modeling (Table S5). Bayesian Lasso estimated parameters in Equations 1,3. Figure 5 presents the TF regulatory network inferred from Equation (3), revealing both antagonistic and synergistic relations; e.g. HOXA1 and TWIST2 suppress PRDM8, whereas HOXA7 promotes it.

**Figure 6:**
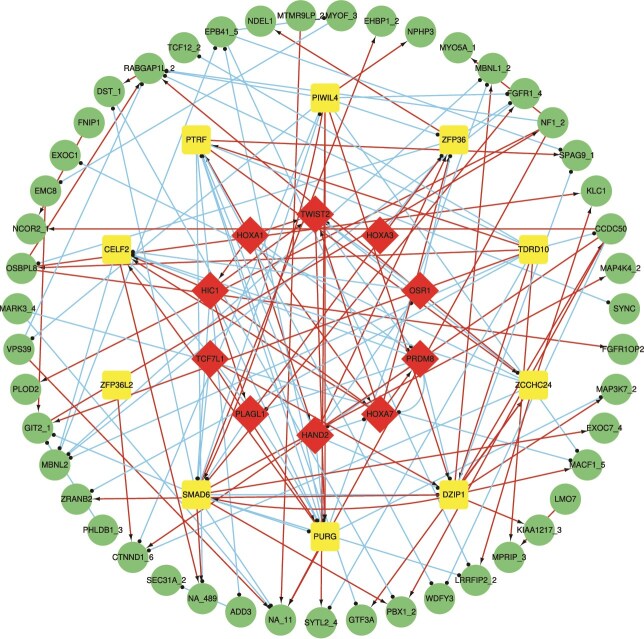
The TF-RBPs-AS regulatory network.

Figure 6 summarizes the integrated TF–RBP–AS network derived from Equations 1,3. The network uncovers hierarchical TF$\to $RBP$\to $AS cascades and both one-to-many and many-to-one control patterns across layers, explaining how coordinated TF–RBP interactions achieve precise splicing regulation.

### Control and analysis of regulatory networks

Table 1 lists key TFs identified by CTC when the constrained set comprises the top 5 TFs (by trend score) and the targets comprise the top 20 AS events; Markov-chain sampling highlights HOXA3, PRDM8, and TWIST2 (frequency $>0.5$). Table S6 reports cytoHubba/MCC results, with HOXA7, HOXA1, PRDM8, and TWIST2 ranked highest.

**Table 1:** The key TFs of the TF–RBP–AS regulatory network

**Key TF**	**Sampling probability**	**Literature support**
HOXA3	1.00	[35, 36]
PRDM8	0.86	[37, 38]
TWIST2	0.83	[39, 40]

**Figure 7:**
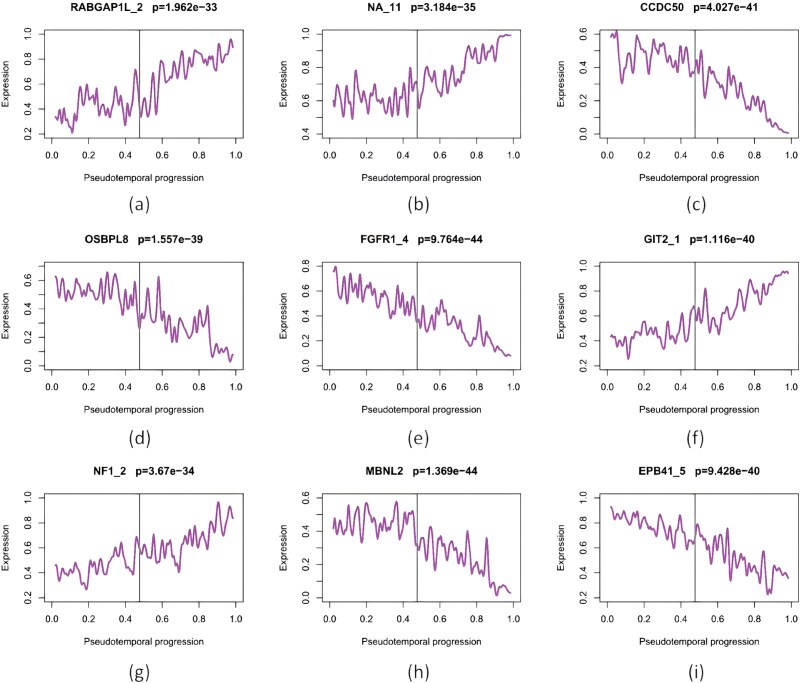
Reconstructed expression dynamics of nine AS events.

Figure 7 shows reconstructed dynamics for nine AS events; all exhibit significant differences (Wilcoxon rank-sum, two-tailed, $P<.05$). Specifically, $\mathrm{RABGAP1L_{2}}$, $\mathrm{NA_{11}}$, $\mathrm{GIT2_{1}}$, and $\mathrm{NF1_{2}}$ are up-regulated, while $\mathrm{CCDC50}$, $\mathrm{OSBPL8}$, $\mathrm{FGFR1_{4}}$, $\mathrm{MBNL2}$, and $\mathrm{EPB41_{5}}$ are down-regulated. Table 2 reports a complementary MCC$\rightarrow $CTC analysis (six TF constraints and AS targets), nominating PRDM8 as a likely master regulator.

**Table 2:** The key TFs of the TF–RBP–AS regulatory network after MCC

**Key TF**	**Sampling probability**	**Literature support**
PRDM8	0.77	[37, 38]

Together, Tables 1 and 2 nominate HOXA3, PRDM8, and TWIST2 as EMT-associated regulators with literature support, suggesting potential therapeutic relevance.

### Biological functional analysis

**Figure 8:**
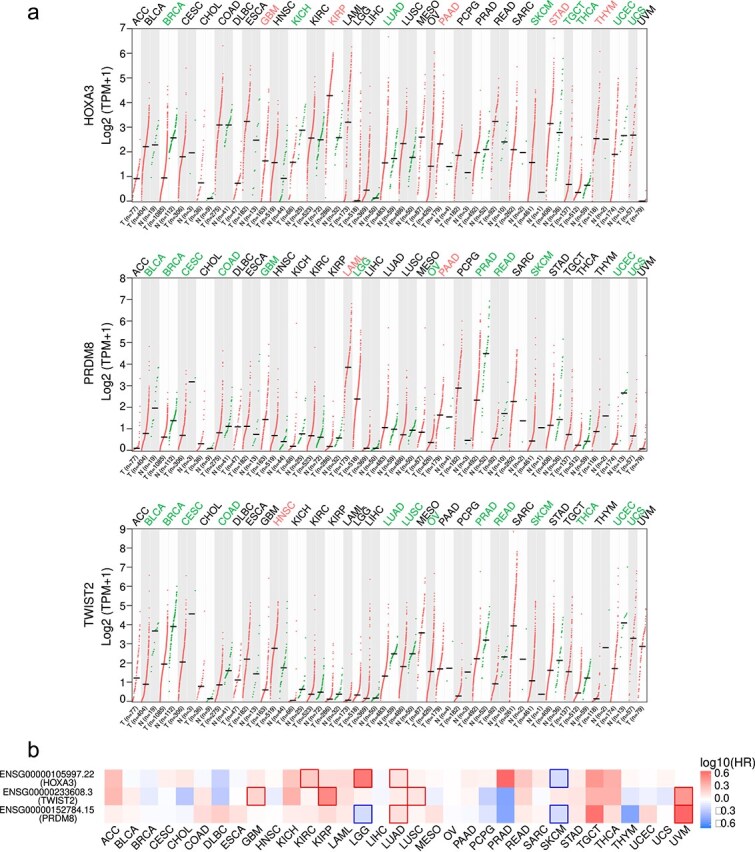
The result of gene expression profile and survival analysis. (a) Differential expression across cancers; (b) overall-survival significance map (HR on $\log _{10}$ scale).

Figure 8a summarizes GEPIA2 expression ($|\log _{2}\mathrm{FC}|>1$, $q<0.01$): HOXA3 is up-regulated in GBM, KIRP, PAAD, STAD, and THYM; PRDM8 in LAML and PAAD; TWIST2 in HNSC. Figure 8b presents overall-survival associations (Mantel–Cox): HOXA3 is high-risk in KIRC, LGG, and LUAD; PRDM8 in LUAD and UVM; TWIST2 in GBM, KIRP, LUSC, and UVM.

**Figure 9:**
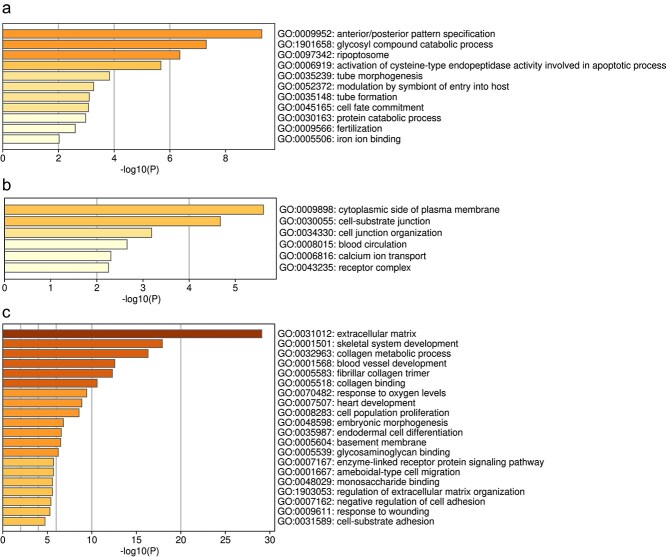
Enrichment analysis of pathways and biological processes was performed using Gene Ontology (GO) Biological Processes. Enriched terms for genes similar to HOXA3 (a), PRDM8 (b), and TWIST2 (c).

Figure 9 reports GO enrichment for genes co-varying with each TF (GEPIA2 selection; Metascape analysis). Genes similar to HOXA3 are enriched for anterior/posterior patterning, glycosyl-compound catabolism, ripoptosome, and apoptosis (Fig. 9a); genes similar to PRDM8 for plasma-membrane cytoplasmic side, cell–substrate junction, and junction organization (Fig. 9b); and genes similar to TWIST2 for extracellular matrix, skeletal development, collagen metabolism, and angiogenesis (Fig. 9c).

### Regulation of alternative splicing events of the CD44 gene in breast cancer

**Figure 10:**
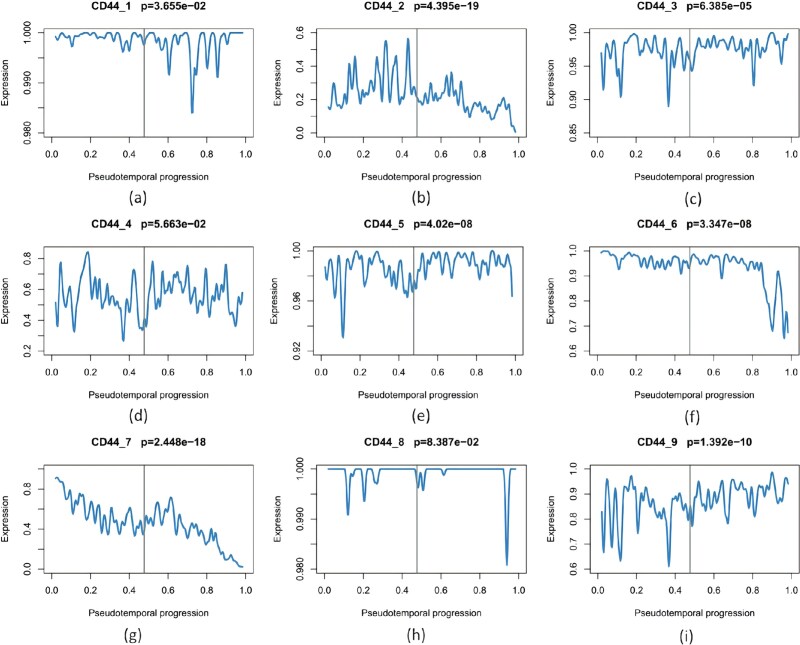
Reconstructed expression dynamics of CD44 gene.

**Figure 11:**
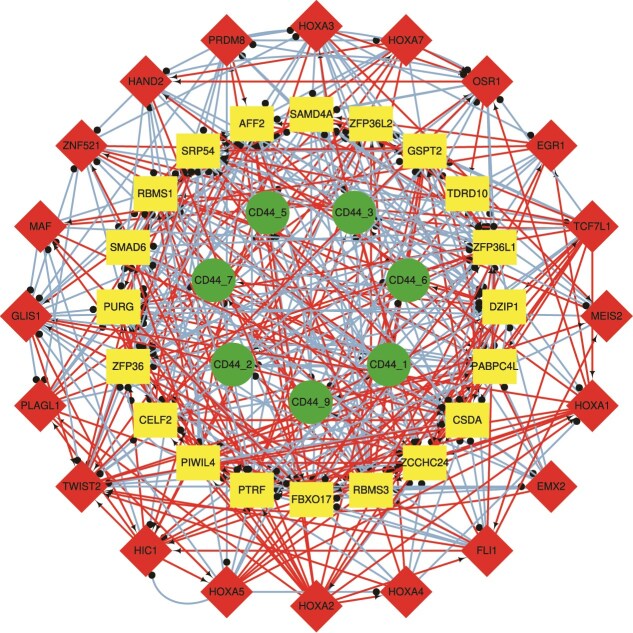
The CD44 regulatory network.

The CD44 pre-mRNA contains 19 exons, nine alternatively spliced; prior work links CD44 splicing to EMT and metastasis [41–43]. Figure 10 shows reconstructed dynamics for nine CD44 AS events; two ($\mathrm{CD44_{4}}$ and $\mathrm{CD44_{8}}$) are not significant ($P>.05$), so network analysis focuses on the remaining seven. Figure 11 displays the CD44 TF/RBP regulatory network built from the top 20 TFs/RBPs selected by trend analysis.

Table 3 summarizes CTC with 20 TF constraints and 7 AS targets, nominating ZNF521 as a key driver. Table 4 reports an MCC-preselected analysis (14 TF constraints and same 7 targets), nominating HIC1. Together, the CD44 case indicates ZNF521 and HIC1 as candidate regulators supported by the literature.

**Table 3:** Key TFs of the CD44 regulatory network

**Key TF**	**Sampling probability**	**Literature support**
ZNF521	0.86	[44]
MAF	0.46	[45]

**Table 4:** The key TFs of the CD44 regulatory network after MCC

**Key TF**	**Sampling probability**	**Literature support**
HIC1	0.65	[46]
PRDM8	0.31	[37]

## Conclusion

In this paper, we introduced CTAS, a network control theory-based framework that integrates pseudotime ordering, trend analysis, sparse directed network inference, and control-theoretic screening to uncover TFs that control AS during EMT. By explicitly modeling the hierarchical TF$\rightarrow $RBP$\rightarrow $AS cascade, CTAS provides a principled way to convert static cross-sectional data into dynamic regulatory insights. Through both simulations and application to a breast cancer EMT cohort, CTAS demonstrated high accuracy in reconstructing pseudotime trajectories and directed networks, and successfully identified biologically supported TFs and subnetworks that regulate AS programs.

The main advantage of CTAS lies in its ability to bridge cross-sectional data and temporal dynamics, thereby offering a novel perspective to prioritize regulators that are most capable of driving splicing changes. This not only deepens our understanding of AS regulation in EMT but also provides experimentally testable hypotheses for cancer biology. Looking forward, CTAS can be extended in several directions. Future work will adapt the framework to more complex trajectories, such as branching processes captured by single-cell transcriptomics, and incorporate perturbation data to strengthen causal inference. In addition, applying CTAS to other biological contexts beyond EMT will help generalize its utility for studying dynamic regulatory programs. Overall, CTAS opens a new avenue for integrating network control theory with omics data to dissect layered regulatory cascades and identify key molecular drivers of AS.

**Key Points**

- This paper proposes a novel framework to identify key regulatory transcription factors (TFs) of alternative splicing(AS) during epithelial–mesenchymal transition based on network control theory.
- This paper develops a new method (CTAS) and compares it with state-of-the-art computational models to illustrate the superiority of the proposed approach. In response to the limitations of existing methods, CTAS reconstructs the hierarchical regulatory relationships among TFs, RNA-binding protein, and AS events to improve regulatory inference.
- CTAS further integrates pseudotime analysis and dynamic network modeling to overcome the limitations of cross-sectional data and uncover latent regulatory mechanisms.
- Biological validation and theoretical analysis are conducted to demonstrate the reliability and interpretability of the proposed framework. Experimental results show that CTAS has strong robustness and generalization ability across simulated and real biological datasets.

## Supplementary Material

S1-Dataset_bbag042

S2-Pseudotime_analysis_bbag042

S3-Temporal_trend_analysis_bbag042

S4-Model__bbag042

S5-Detailed_algorithm_bbag042

S6-Robustness_analysis_bbag042

S7-Testing_method_with_a_synthetic_dataset_bbag042

supp_table_bbag042

## Data Availability

The code and datasets of CTAS are available from https://github.com/Yangsong-He/CTAS.

## References

[1] Valastyan S, Weinberg RA. Tumor metastasis: molecular insights and evolving paradigms. *Cell* 2011; 147:275–92. 22000009 10.1016/j.cell.2011.09.024 PMC3261217

[2] Kalluri R, Weinberg RA. The basics of epithelial-mesenchymal transition. *J Clin Invest* 2009; 119:1420–8. 10.1172/JCI39104 19487818 PMC2689101

[3] Nieto MA, Huang RYJ, Jackson RA. et al. EMT: 2016. *Cell* 2016; 166:21–45. 10.1016/j.cell.2016.06.028 27368099

[4] Zeisberg M, Neilson EG. Biomarkers for epithelial-mesenchymal transitions. *J Clin Invest* 2009; 119:1429–37. 10.1172/JCI36183 19487819 PMC2689132

[5] Mani SA, Yang J, Brooks M. et al. Mesenchyme Forkhead 1 (FOXC2) plays a key role in metastasis and is associated with aggressive basal-like breast cancers. *Proc Natl Acad Sci USA* 2007; 104:10069–74. 17537911 10.1073/pnas.0703900104 PMC1891217

[6] Yang J, Mani SA, Donaher JL. et al. Twist, a master regulator of morphogenesis, plays an essential role in tumor metastasis. *Cell* 2004; 117:927–39. 15210113 10.1016/j.cell.2004.06.006

[7] Roy B, Haupt LM, Griffiths LR. Alternative splicing (AS) of genes as an approach for generating protein complexity. *Curr Genomics* 2013; 14:182–94. 10.2174/1389202911314030004 24179441 PMC3664468

[8] Pan Q, Shai O, Lee LJ. et al. Deep surveying of alternative splicing complexity in the human transcriptome by high-throughput sequencing. *Nat Genet* 2008; 40:1413–5. 18978789 10.1038/ng.259

[9] Nilsen TW, Graveley BR. Expansion of the eukaryotic proteome by alternative splicing. *Nature* 2010; 463:457–63. 10.1038/nature08909 20110989 PMC3443858

[10] Fu XD, Ares MJr. Context-dependent control of alternative splicing by RNA-binding proteins. *Nat Rev Genet* 2014; 15:689–701. 25112293 10.1038/nrg3778 PMC4440546

[11] Ule J, Blencowe BJ. Alternative splicing regulatory networks: functions, mechanisms, and evolution. *Mol Cell* 2019; 76:329–45. 10.1016/j.molcel.2019.09.017 31626751

[12] Lambert SA, Jolma A, Campitelli LF. et al. The human transcription factors. *Cell* 2018; 172:650–65. 10.1016/j.cell.2018.01.029 29425488 PMC12908702

[13] Girardot M, Bayet E, Maurin J. et al. SOX9 has distinct regulatory roles in alternative splicing and transcription. *Nucleic Acids Res* 2018; 46:9106–18. 10.1093/nar/gky553 29901772 PMC6158501

[14] Marku M, Pancaldi V. From time-series transcriptomics to gene regulatory networks: a review on inference methods. *PLoS Comput Biol* 2023; 19:e1011254. 10.1371/journal.pcbi.1011254 37561790 PMC10414591

[15] Kauffman SA . Metabolic stability and epigenesis in randomly constructed genetic nets. *J Theor Biol* 1969; 22:437–67. 5803332 10.1016/0022-5193(69)90015-0

[16] Akutsu T, Miyano S, Kuhara S. Identification of genetic networks from a small number of gene expression patterns under the Boolean network model. *Biocomputing’99.* 1999;4:17–28. 10.1142/9789814447300_0003 10380182

[17] Chen T, He HL, Church GM. Modeling gene expression with differential equations. *Biocomputing’99.* 1999;4:29–40. 10380183

[18] Cao J, Qi X, Zhao H. Modeling gene regulation networks using ordinary differential equations. *Methods Mol Biol* 2012;802:185–97. 10.1007/978-1-61779-400-1_12 22130881

[19] Mihajlovic V, Petkovic M. Dynamic Bayesian networks: a state of the art. *Technical Report* University of Twente; 2001.

[20] Suter P, Kuipers J, Beerenwinkel N. Discovering gene regulatory networks of multiple phenotypic groups using dynamic Bayesian networks. *Brief Bioinform* 2022; 23:bbac219. 10.1093/bib/bbac219 PMC9294428 35679575

[21] Chan TE, Stumpf MPH, Babtie AC. Gene regulatory network inference from single-cell data using multivariate information measures. *Cell Systems* 2017; 5:251–267.e3. 10.1016/j.cels.2017.08.014 28957658 PMC5624513

[22] Huynh-Thu VA, Irrthum A, Wehenkel L. et al. Inferring regulatory networks from expression data using tree-based methods. *PLoS One* 2010; 5:e12776. 10.1371/journal.pone.0012776 20927193 PMC2946910

[23] Liu YY, Slotine JJ, Barabási AL. Controllability of complex networks. *Nature* 2011; 473:167–73. 10.1038/nature10011 21562557

[24] Li M, Gao H, Wang J. et al. Control principles for complex biological networks. *Brief Bioinform* 2019; 20:2253–66. 10.1093/bib/bby088 30239577

[25] Hu Y, Chen C, Ding Y. et al. Optimal control nodes in disease-perturbed networks as targets for combination therapy. *Nat Commun* 2019; 10:2180. 10.1038/s41467-019-10215-y 31097707 PMC6522545

[26] Fiedler B, Mochizuki A, Kurosawa G. et al. Dynamics and control at feedback vertex sets. I: Informative and determining nodes in regulatory networks. *J Dyn Differ Equ* 2013; 25:563–604.

[27] Mochizuki A, Fiedler B, Kurosawa G. et al. Dynamics and control at feedback vertex sets. II: A faithful monitor to determine the diversity of molecular activities in regulatory networks. *J Theor Biol* 2013; 335:130–46. 23774067 10.1016/j.jtbi.2013.06.009

[28] Zañudo JGT, Yang G, Albert R. Structure-based control of complex networks with nonlinear dynamics. *Proc Natl Acad SciUSA* 2017; 114:7234–9. 10.1073/pnas.1617387114 PMC5514702 28655847

[29] Nacher JC, Akutsu T. Dominating scale-free networks with variable scaling exponent: heterogeneous networks are not difficult to control. *New J Phys* 2012; 14:073005.

[30] Guo WF, Zhang SW, Zeng T. et al. A novel network control model for identifying personalized driver genes in cancer. *PLoS Comput Biol* 2019; 15:e1007520. 10.1371/journal.pcbi.1007520 31765387 PMC6901264

[31] Sun X, Zhang J, Nie Q. Inferring latent temporal progression and regulatory networks from cross-sectional transcriptomic data of cancer samples. *PLoS Comput Biol* 2021; 17:e1008379. 10.1371/journal.pcbi.1008379 33667222 PMC7968745

[32] Sun L, Qiu Y, Ching WK. et al. PCB: a pseudotemporal causality-based Bayesian approach to identify EMT-associated regulatory relationships of AS events and RBPs during breast cancer progression. *PLoS Comput Biol* 2023; 19:e1010939. 10.1371/journal.pcbi.1010939 36930678 PMC10057809

[33] Qiu Y, Lyu J, Dunlap M. et al. A combinatorially regulated RNA splicing signature predicts breast cancer EMT states and patient survival. *RNA.* 2020; 26:1257–67. 10.1261/rna.074187.119 32467311 PMC7430667

[34] Guo WF, Zhang SW, Wei ZG. et al. Constrained target controllability of complex networks. *J Stat Mech Theory Exp* 2017; 2017:063402.

[35] Paço A, de Bessa A, Garcia S. et al. Roles of the HOX proteins in cancer invasion and metastasis. *Cancers* 2020; 13:10. 33375038 10.3390/cancers13010010 PMC7792759

[36] Tapia-Carrillo D, Tovar H, Velazquez-Caldelas TE. et al. Master regulators of signaling pathways: an application to the analysis of gene regulation in breast cancer. *Front Genet* 2019; 10:1180. 31850059 10.3389/fgene.2019.01180 PMC6902642

[37] Wu X, Miao J, Jiang J. et al. Analysis of methylation profiling data of hyperplasia and primary and metastatic endometrial cancers. *Eur J Obstet Gynecol Reprod Biol* 2017; 217:161–6. 10.1016/j.ejogrb.2017.08.036 28910750

[38] Mzoughi S, Tan YX, Low D. et al. The role of PRDMs in cancer: one family, two sides. *Curr Opin Genet Dev* 2016; 36:83–91. 10.1016/j.gde.2016.03.009 27153352

[39] Fang X, Cai Y, Liu J. et al. TWIST2 contributes to breast cancer progression by promoting an epithelial–mesenchymal transition and cancer stem-like cell self-renewal. *Oncogene* 2011; 30:4707–20. 10.1038/onc.2011.181 21602879

[40] Mao Y, Xu J, Li Z. et al. The role of nuclear $\beta $-catenin accumulation in the TWIST2-induced ovarian cancer EMT. *PLoS One* 2013; 8:e78200. 10.1371/journal.pone.0078200 24244294 PMC3823872

[41] Brown RL, Reinke LM, Damerow MS. et al. CD44 splice isoform switching in human and mouse epithelium is essential for epithelial-mesenchymal transition and breast cancer progression. *J Clin Invest* 2011; 121:1064–74. 21393860 10.1172/JCI44540 PMC3049398

[42] Reinke LM, Xu Y, Cheng C. Snail represses the splicing regulator epithelial splicing regulatory protein 1 to promote epithelial-mesenchymal transition. *J Biol Chem* 2012; 287:36435–42. 10.1074/jbc.M112.397125 22961986 PMC3476309

[43] Zhao P, Xu Y, Wei Y. et al. The CD44s splice isoform is a central mediator for invadopodia activity. *J Cell Sci* 2016; 129:1355–65. 10.1242/jcs.171959 26869223 PMC6518308

[44] Scicchitano S, Montalcini Y, Lucchino V. et al. Enhanced ZNF521 expression induces an aggressive phenotype in human ovarian carcinoma cell lines. *PLoS One* 2022; 17:e0274785. 10.1371/journal.pone.0274785 36191006 PMC9529122

[45] Nasrazadani A, Gomez Marti JL, Hyder T. et al. Investigation of a genomic signature for transcription factor MAF gene amplification and lack of bisphosphonate benefit in early breast cancer. *Cancer Res* 2022; 82:559–9.

[46] Wang Y, Weng X, Wang L. et al. HIC1 deletion promotes breast cancer progression by activating tumor cell/fibroblast crosstalk. *J Clin Invest* 2018; 128:5235–50. 30204129 10.1172/JCI99974 PMC6264654

