# Supplementary material for: CTAS: a network control theory-based approach to identify key regulatory TFs of AS events during epithelial–mesenchymal transition
Source: Brief Bioinform. 2026 Feb 10;27(1):bbag042. doi: 10.1093/bib/bbag042 (PMC12888823; doi:10.1093/bib/bbag042)
Supplement: S1-Dataset_bbag042 [file s1-dataset_bbag042.pdf]

Dataset introduction

Epithelial and mesenchymal tumors were then expected to be distinguished from the original TCGA-BRCA samples. EM transition score was calculated based on the gene expression differences between the epithelial cell marker, E-cadherin (CDH1), and mesenchymal cell marker, vimentin (VIM), which exhibit high expression in epithelial and mesenchymal states, respectively. Based on EM transition score, the samples were classified into epithelial and mesenchymal groups. Samples with the EM transition score greater than one standard deviation above the mean were categorized as mesenchymal, and those with the EM transition lower than one standard deviation below the mean were classified as epithelial. Ultimately, 143 epithelial samples and 157 mesenchymal samples were identified from the 1215 TCGA-BRCA samples.

Known alternative splicing events could be classified using an annotated set of splicing events provided by the splicing analysis tool mixture-of-isoforms (MISO) model downloaded from (http://hollywood.mit.edu.eproxy.lib.hku.hk/burgelab/miso/). Based on the above analysis, a comprehensive list of 15753 annotated cassette exons events was created for the 300 samples (143 epithelial samples and 157 mesenchymal samples). The data consist of PSI values for alternative splicing events in cancer patient samples. PSI values use ratios to quantify the expression of AS events. For example, if a gene has three splice variants, and assuming that variant AS1 accounts for 50% of the total expression of the gene, variant AS2 accounts for 30%, and variant AS3 accounts for 20%, the expression of the three AS events of the gene is the total expression of the gene multiplied by the corresponding ratios. Meanwhile, we collected the gene expressions of 1532 RNA-binding proteins in a recent census of human RNA-binding proteins for all breast cancer (BRCA)-associated samples. And we collected the gene expressions of 1500 transcription factors of all breast cancer (BRCA)-associated samples according to a human transcription factors list.
