# Supplementary material for: CTAS: a network control theory-based approach to identify key regulatory TFs of AS events during epithelial–mesenchymal transition
Source: Brief Bioinform. 2026 Feb 10;27(1):bbag042. doi: 10.1093/bib/bbag042 (PMC12888823; doi:10.1093/bib/bbag042)
Supplement: S2-Pseudotime_analysis_bbag042 [file s2-pseudotime_analysis_bbag042.pdf]

PSEUDOTIME ANALYSIS

We used cross-sectional data from the TCGA-BRCA cohort [1], including 143 epithelial and 157 mesenchymal specimens among 1,215 breast cancer samples. Let $\mathcal{S} = \{x_1, \ldots, x_n\}$ be the breast-cancer cohort under study. For each sample $x \in \mathcal{S}$ we extract a size-p expression vector $\boldsymbol{T}_x \in \mathbb{R}^p$ comprising all transcription-factor (TF) genes after library-size normalisation and log transformation. Every patient is labeled by a binary stage indicator $\ell_x \in \{1, 2\}$, where $\ell_x = 1$ denotes an epithelial specimen and $\ell_x = 2$ marks a mesenchymal specimen. To adapt the subsequent kernel to heterogeneous sampling density, the local scale parameter $h_x = \operatorname{dist}\big(\boldsymbol{T}_x, k\text{th-nearest neighbour of } \boldsymbol{T}_x\big)$ is computed in the TF space ($k = 3$ throughout this work). Then we propose algorithm for pseudotime analysis [2] below.

a) Stage–aware similarity kernel: With the above definitions we construct an affinity

$$K(x, y) \;=\; \exp\!\Big\{-\frac{\|\boldsymbol{T}_x - \boldsymbol{T}_y\|_2^2}{h_x^2 + h_y^2}\Big\} \;\times\; \exp\!\big(-\delta_{\ell_x \neq \ell_y}\big), \tag{1}$$

where $\delta_{\ell_x \neq \ell_y}$ is 1 if the two samples belong to different EMT stages and 0 otherwise. Thus inter-stage pairs are down-weighted exponentially, whereas the bandwidth $h_x^2 + h_y^2$ rescales Euclidean distance according to local density.

b) Diffusion normalisation: A two-step normalisation is applied to remove sampling-density bias.

First, define the degree function $D(x) = \sum_{z \in \mathcal{S}} K(x, z)$, which measures the total similarity between sample x and all other samples in the cohort. This reflects the local sampling density around x in the expression manifold.

Then construct the density-normalised kernel matrix:

$$H_{xy} = \frac{K(x, y)}{D(x) D(y)}, \tag{2}$$

which reduces the influence of densely sampled regions by rescaling the affinities.

Second, define $E(x) = \sum_{z \in \mathcal{S}} H_{xz}$ as the row sum of H, capturing the total adjusted affinity of x under the density-normalised kernel.

The row-stochastic diffusion matrix is then computed by

$$P_{xy} = \frac{H_{xy}}{\sqrt{E(x) E(y)}}, \tag{3}$$

ensuring that P is both symmetric and properly normalised for downstream diffusion-based analysis.

c) Global diffusion coordinates: Let ψ_0 be the principal eigenvector of P (eigenvalue 1). Following the heat-kernel formulation, the accumulated diffusion propagator is

$$Q = \left[I - \left(P - \psi_0\psi_0^\top\right)\right]^\dagger - I, \tag{4}$$

where † denotes the Moore–Penrose pseudoinverse. Row $Q(x,\cdot)$ provides a global coordinate for sample x capturing multi-step connectivity in the TF manifold.

d) Ordering along the EMT axis: The diffusion distance $d(x,y) = \|Q(x,\cdot) - Q(y,\cdot)\|_2$ is used to build a one-dimensional timeline.

Define the pseudotime distance (PTD) by $d(x,y) = \|Q(x,\cdot) - Q(y,\cdot)\|_2$. Choose a random mesenchymal sample x_{ref} and set the root

$$x_\star = \arg\max_{x\in\mathcal{E}} \mathrm{PTD}(x, x_{\text{ref}}). \tag{5}$$

Each sample then receives a pseudotime score

$$s_x = \mathrm{PTD}(x, x_\star), \tag{6}$$

and the cohort is ordered increasingly by s_x.

e) Propagation to other omics layers: The ordering derived from TFs is imposed verbatim on the RBP matrix and on the PSI matrix of alternative-splicing events, thereby mapping every molecular layer onto the same latent EMT progression axis.

References

[1] Qiu Y, Lyu J, Dunlap M, et al. A combinatorially regulated RNA splicing signature predicts breast cancer EMT states and patient survival. RNA. 2020;26(9):1257–1267.

[2] Sun X, Zhang J, Nie Q. Inferring latent temporal progression and regulatory networks from cross-sectional transcriptomic data of cancer samples. PLoS Computational Biology. 2021;17(3):e1008379.
