# Supplementary material for: CTAS: a network control theory-based approach to identify key regulatory TFs of AS events during epithelial–mesenchymal transition
Source: Brief Bioinform. 2026 Feb 10;27(1):bbag042. doi: 10.1093/bib/bbag042 (PMC12888823; doi:10.1093/bib/bbag042)
Supplement: S3-Temporal_trend_analysis_bbag042 [file s3-temporal_trend_analysis_bbag042.pdf]

TEMPORAL TREND ANALYSIS

Following the inference of pseudotime trajectories, we aim to identify transcription factors (TFs), RNA-binding proteins (RBPs), and alternative splicing (AS) events that are dynamically regulated during epithelial–mesenchymal transition (EMT)[1]. To achieve this, we perform a temporal trend analysis that quantifies the degree to which each molecular component exhibits systematic change along the pseudotemporal axis.

For each gene or splicing event v, let $x_v = \left[x_v^{(1)}, x_v^{(2)}, \ldots, x_v^{(n)}\right]$ denote its expression values across n samples ordered by pseudotime. We fit a linear model:

$$x_v^{(i)} = \alpha_v + \beta_v s^{(i)} + \varepsilon^{(i)}, \tag{1}$$

where $s^{(i)}$ is the pseudotime coordinate of sample i, β_v captures the linear trend over time, and $\varepsilon^{(i)}$ denotes residual variation after detrending.

To robustly quantify temporal dynamics, we define the trend score as:

$$\mathrm{Trend}(v) = \left| \frac{\beta_v}{\sigma_{\mathrm{resid}}(v)} \right|, \tag{2}$$

where $\sigma_{\mathrm{resid}}(v)$ is the standard deviation of the residuals from the fitted linear model. This ratio effectively penalizes random fluctuations while emphasizing consistent upward or downward trends.

A higher trend score indicates stronger temporal regulation of the gene or event during the EMT process. All transcription factors, RNA-binding proteins, and alternative splicing events are independently ranked by their trend scores. Top-ranking components are selected for subsequent modeling of the hierarchical regulatory cascade involving TF $\rightarrow$ RBP $\rightarrow$ AS.

This scoring method provides an interpretable and scalable approach to extract temporally informative molecular signals from cross-sectional data that has been temporally reconstructed using pseudotime analysis.

REFERENCES

[1] Sun L, Qiu Y, Ching WK, et al. PCB: A pseudotemporal causality-based Bayesian approach to identify EMT-associated regulatory relationships of AS events and RBPs during breast cancer progression. PLOS Computational Biology. 2023;19(3):e1010939.
