# Supplementary material for: CTAS: a network control theory-based approach to identify key regulatory TFs of AS events during epithelial–mesenchymal transition
Source: Brief Bioinform. 2026 Feb 10;27(1):bbag042. doi: 10.1093/bib/bbag042 (PMC12888823; doi:10.1093/bib/bbag042)
Supplement: S4-Model__bbag042 [file s4-model__bbag042.pdf]

MODEL OF TF-RBP-AS EVENT REGULATORY RELATIONSHIPS

The TF-RBP-AS regulatory relationships can be modeled using the following dynamical system,

$$\frac{\mathrm{d}X_i(s)}{\mathrm{d}s} = \sum_{j\neq i} a_{ij}X_i(s)\cdot X_j(s) + \sum_{l=1}^{M} b_{il}X_i(s)\cdot Y_l(s) - d_iX_i(s), \tag{1}$$

$$\frac{\mathrm{d}Y_l(s)}{\mathrm{d}s} = \sum_{k\neq l} c_{lk}Y_l(s)\cdot Y_k(s) + \sum_{p=1}^{H} e_{lp}Y_l(s)\cdot Z_p(s) - d_l'Y_l(s), \tag{2}$$

$$\frac{\mathrm{d}Z_p(s)}{\mathrm{d}s} = \sum_{p\neq q} g_{pq}Z_p(s)\cdot Z_q(s) - d_p''Z_p(s), \tag{3}$$

where $X_i(s)$, $Y_l(s)$ and $Z_p(s)$ represent the expression level of AS event $i,\ i=1,2,\dots,N$, RNA-binding protein $l,\ l=1,2,\dots,M$ and TF $p,\ p=1,2,\dots,H$ in breast cancer with pseudo-time progression status s, respectively. Moreover, a_{ij} is the dynamic regulatory coefficient from AS event j to AS event i, where $i\neq j$ and $i,j=1,2,\dots,N$ b_{il} is the dynamic regulatory coefficient from RNA-binding protein l to AS event i, where $i=1,2,\dots,N$ and $l=1,2,\dots,M$; c_{lk} is the dynamic regulatory coefficient from RNA-binding protein k to RNA-binding protein l, where $l\neq k$ and $l,k=1,2,\dots,M$; e_{lp} is the dynamic regulatory coefficient from TF p to RNA-binding protein l, where $l=1,2,\dots,M$ and $p=1,2,\dots,H$; g_{pq} is the dynamic regulatory coefficient from TF q to TF p, where $p\neq q$ and $p,q=1,2,\dots,H$, and d_i is the self-degradation rate of AS event i; d_l' is the self-degradation rate of RBP l, d_p'' is the self-degradation rate of TF p.

The details of model assumption and derivative are provided as follows.

Let $X_i(t,s)$, $Y_l(t,s)$ and $Z_p(t,s)$ represent the expression level of AS event $i,\ i=1,2,\dots,N$, RNA-binding protein $l,\ l=1,2,\dots,M$ and TF $p,\ p=1,2\dots H$ at time t in breast cancer with pseudotime progression status s, respectively. Assume cancer progression is an irreversible process over time so that $s=\varphi(t)$ is a strictly monotonic increasing function of t, i.e., $\varphi'(t)=\delta>0$. As such, $s=\varphi(t)$ has an inverse function $t=\varphi^{-1}(s)$ and $\frac{\mathrm{d}t}{\mathrm{d}s}=\frac{1}{\varphi'(t)}=\frac{1}{\delta}$.

The change rates of $X_i(t,s)$, $Y_l(t,s)$ and $Z_p(t,s)$ after a small progression period Δs can be modeled by the following two difference equations:

$$\begin{aligned}&\frac{X_i(\varphi^{-1}(s+\Delta s),s+\Delta s)-X_i(t,s)}{\Delta s}\\ =&\sum_{j\neq i} a_{ij}X_i(t,s)\cdot X_j(t,s) + \sum_{l=1}^{M} b_{il}X_i(t,s)\cdot Y_l(t,s) - d_iX_i(t,s),\end{aligned} \tag{4}$$

$$\frac{Y_l(\varphi^{-1}(s+\Delta s), s+\Delta s) - Y_l(t,s)}{\Delta s} \\ = \sum_{k\neq l} c_{lk} Y_l(t,s)\cdot Y_k(t,s) + \sum_{p=1}^{H} e_{lp} Y_l(t,s)\cdot Z_p(t,s) - d_l' Y_l(t,s), \tag{5}$$

$$\frac{Z_p(\varphi^{-1}(s+\Delta s), s+\Delta s) - Z_p(t,s)}{\Delta s} = \sum_{p\neq q} g_{pq} Z_p(t,s)\cdot Z_q(t,s) - d_p'' Z_p(t,s), \tag{6}$$

where a_{ij} is the dynamic regulatory coefficient from AS event j to AS event i, where $i \neq j$, b_{il} is the dynamic regulatory coefficient from RNA-binding protein l to AS event i, c_{lk} is the dynamic regulatory coefficient from RNA-binding protein k to RNA-binding protein l, where $l \neq k$, e_{lp} is the dynamic regulatory coefficient from TF p to RNA-binding protein l, g_{pq} is the dynamic regulatory coefficient from TF q to TF p, where $p \neq q$, and d_i, d_l', d_p'' is the self-degradation rate of AS event i, RBP l, d_p'' and TF p, respectively.

As $\Delta s \to 0$, we obtain the following progression-structured model in the form of partial differential equations (PDEs),

$$\frac{\partial X_i}{\partial t}\cdot(\varphi^{-1}(s))' + \frac{\partial X_i}{\partial s} = \sum_{j\neq i} a_{ij} X_i(t,s)\cdot X_j(t,s) + \sum_{l=1}^{M} b_{il} X_i(t,s)\cdot Y_l(t,s) - d_i X_i(t,s), \tag{7}$$

$$\frac{\partial Y_l}{\partial t}\cdot(\varphi^{-1}(s))' + \frac{\partial Y_l}{\partial s} = \sum_{k\neq l} c_{lk} Y_l(t,s)\cdot Y_k(t,s) + \sum_{p=1}^{H} e_{lp} Y_l(t,s)\cdot Z_p(t,s) - d_l' Y_l(t,s), \tag{8}$$

$$\frac{\partial Z_p}{\partial t}\cdot(\varphi^{-1}(s))' + \frac{\partial Z_p}{\partial s} = \sum_{p\neq q} g_{pq} Z_p(t,s)\cdot Z_q(t,s) - d_p'' Z_p(t,s), \tag{9}$$

Since gene regulations or biochemical reactions are notably faster than cancer progression, we could assume that, in the above equations, $X_i(t,s)$, $Y_l(t,s)$ and $Z_p(t,s)$ quickly approach its steady-state $\bar{X}_i(s)$, $\bar{Y}_l(s)$ and $\bar{Z}_p$ as s changes, respectively, that is, $\frac{\partial \bar{X}_i(s)}{\partial t} = 0$, $\frac{\partial \bar{Y}_l(s)}{\partial t} = 0$ and $\frac{\partial \bar{Z}_p(s)}{\partial t} = 0$. Therefore, we have the following ordinary differential equations (ODEs):

$$\frac{\mathrm{d}\bar{X}_i(s)}{\mathrm{d}s} = \frac{\partial \bar{X}_i(s)}{\partial s} = \sum_{j\neq i} a_{ij}\bar{X}_i(s)\cdot \bar{X}_j(s) + \sum_{l=1}^{M} b_{il}\bar{X}_i(s)\cdot \bar{Y}_l(s) - d_i\bar{X}_i(s), \tag{10}$$

$$\frac{\mathrm{d}\bar{Y}_l(s)}{\mathrm{d}s} = \frac{\partial \bar{Y}_l(s)}{\partial s} = \sum_{k\neq l} c_{lk}\bar{Y}_l(s)\cdot \bar{Y}_k(s) + \sum_{l=1}^{M} e_{lp}\bar{Y}_l(s)\cdot \bar{Z}_p(s) - d_l'\bar{Y}_l(s), \tag{11}$$

$$\frac{\mathrm{d}\bar{Z}_p(s)}{\mathrm{d}s} = \frac{\partial \bar{Z}_p(s)}{\partial s} = \sum_{p\neq q} g_{pq}\bar{Z}_p(s)\cdot \bar{Z}_q(s) - d_p''\bar{Z}_p(s). \tag{12}$$

For simplicity, when there is no ambiguity, we write $\bar{X}_i(s)$ as $X_i(s)$, $\bar{Y}_l(s)$ as $Y_l(s)$ and $\bar{Z}_p(s)$ as $Z_p(s)$. (10), (11) and (12) can therefore be rewritten as

$$\frac{\mathrm{d}X_i(s)}{\mathrm{d}s} = \sum_{j\neq i} a_{ij} X_i(s)\cdot X_j(s) + \sum_{l=1}^{M} b_{il} X_i(s)\cdot Y_l(s) - d_i X_i(s), \tag{13}$$

$$\frac{\mathrm{d}Y_l(s)}{\mathrm{d}s} = \sum_{k\neq l} c_{lk} Y_l(s)\cdot Y_k(s) + \sum_{p=1}^{H} e_{lp} Y_l(s)\cdot Z_p(s) - d'_l Y_l(s), \tag{14}$$

$$\frac{\mathrm{d}Z_p(s)}{\mathrm{d}s} = \sum_{p\neq q} g_{pq} Z_p(s)\cdot Z_q(s) - d''_p Z_p(s), \tag{15}$$

Take $m+1$ points $s_i = s(r_i)$ from the smoothed progression trajectory $s(r)$, where $r_i = \frac{i}{m},\ i = 0, 1, \ldots, m$. We approximate

$$\frac{\mathrm{d}X_i(s_t)}{\mathrm{d}s} \approx \frac{X_i(s_{t+1}) - X_i(s_t)}{s_{t+1} - s_t}, \quad \frac{\mathrm{d}Y_l(s_t)}{\mathrm{d}s} \approx \frac{Y_l(s_{t+1}) - Y_l(s_t)}{s_{t+1} - s_t}, \quad \frac{\mathrm{d}Z_p(s_t)}{\mathrm{d}s} \approx \frac{Z_p(s_{t+1}) - Z_l(s_t)}{s_{t+1} - s_t}, \tag{16}$$

and denote

$$U_{it} = \frac{X_i(s_{t+1}) - X_i(s_t)}{s_{t+1} - s_t}, \quad V_{lt} = \frac{Y_l(s_{t+1}) - Y_l(s_t)}{s_{t+1} - s_t}, \quad W_{pt} = \frac{Z_p(s_{t+1}) - Z_p(s_t)}{s_{t+1} - s_t}, \tag{17}$$

where $s_{t+1} - s_t$ is sufficiently small (since m could be chosen large enough). Therefore, the above continuous model (i.e., (1), (2) and (3)) can be discretized and rewritten as

$$U_{it} \approx \sum_{j\neq i} a_{ij} X_i(s_t)\cdot X_j(s_t) + \sum_{l=1}^{M} b_{il} X_i(s_t)\cdot Y_l(s_t) - d_i X_i(s_t), \tag{18}$$

$$V_{lt} \approx \sum_{k\neq l} c_{lk} Y_l(s_t)\cdot Y_k(s_t) + \sum_{p=1}^{H} e_{lp} Y_l(s_t)\cdot Z_p(s_t) - d'_l Y_l(s_t), \tag{19}$$

$$W_{pt} \approx \sum_{p\neq q} g_{pq} Z_p(s_t)\cdot Z_q(s_t) - d''_p Z_p(s_t), \tag{20}$$

where $t = 0, 1, \ldots, m-1$.

We then denote

$$U_i = (U_{i0}, \ldots, U_{it}, \ldots, U_{i,m-1})^{\mathrm{T}}, \tag{21}$$

$$V_l = (V_{l0}, \ldots, V_{lt}, \ldots, V_{l,m-1})^{\mathrm{T}}, \tag{22}$$

$$W_p = (W_{p0}, \ldots, W_{pt}, \ldots, W_{p,m-1})^{\mathrm{T}}, \tag{23}$$

$$A_i = (a_{i1}, a_{i2}, \ldots, a_{iN}, b_{i1}, b_{i2}, \ldots, b_{iM}, -d_i)^{\mathrm{T}}, \quad a_{ii} = 0, \tag{24}$$

$$C_l = (c_{l1}, c_{l2}, \ldots, c_{lM}, e_{l1}, e_{l2}, \ldots, e_{lH}, -d'_l)^{\mathrm{T}}, \quad c_{ll} = 0, \tag{25}$$

$$G_p = (g_{p1}, g_{p2}, \ldots, g_{pH}, -d''_p)^{\mathrm{T}}, \quad g_{pp} = 0, \tag{26}$$

and

$$X^{(i)} = \begin{bmatrix} X_i(s_0)X_1(s_0) & X_i(s_1)X_1(s_1) & \cdots & X_i(s_{m-1})X_1(s_{m-1}) \\ X_i(s_0)X_2(s_0) & X_i(s_1)X_2(s_1) & \cdots & X_i(s_{m-1})X_2(s_{m-1}) \\ \cdots & \cdots & \cdots & \cdots \\ X_i(s_0)X_N(s_0) & X_i(s_1)X_N(s_1) & \cdots & X_i(s_{m-1})X_N(s_{m-1}) \\ X_i(s_0)Y_1(s_0) & X_i(s_1)Y_1(s_1) & \cdots & X_i(s_{m-1})Y_1(s_{m-1}) \\ X_i(s_0)Y_2(s_0) & X_i(s_1)Y_2(s_1) & \cdots & X_i(s_{m-1})Y_2(s_{m-1}) \\ \cdots & \cdots & \cdots & \cdots \\ X_i(s_0)Y_M(s_0) & X_i(s_1)Y_M(s_1) & \cdots & X_i(s_{m-1})Y_M(s_{m-1}) \\ X_i(s_0) & X_i(s_1) & \cdots & X_i(s_{m-1}) \end{bmatrix}^{\mathrm{T}}, \tag{27}$$

$$Y^{(i)} = \begin{bmatrix} Y_i(s_0)Y_1(s_0) & Y_i(s_1)Y_1(s_1) & \cdots & Y_i(s_{m-1})Y_1(s_{m-1}) \\ Y_i(s_0)Y_2(s_0) & Y_i(s_1)Y_2(s_1) & \cdots & Y_i(s_{m-1})Y_2(s_{m-1}) \\ \cdots & \cdots & \cdots & \cdots \\ Y_i(s_0)Y_N(s_0) & Y_i(s_1)Y_N(s_1) & \cdots & Y_i(s_{m-1})Y_N(s_{m-1}) \\ Y_i(s_0)Z_1(s_0) & Y_i(s_1)Z_1(s_1) & \cdots & Y_i(s_{m-1})Z_1(s_{m-1}) \\ Y_i(s_0)Z_2(s_0) & Y_i(s_1)Z_2(s_1) & \cdots & Y_i(s_{m-1})Z_2(s_{m-1}) \\ \cdots & \cdots & \cdots & \cdots \\ Y_i(s_0)Z_H(s_0) & Y_i(s_1)Z_H(s_1) & \cdots & Y_i(s_{m-1})Z_H(s_{m-1}) \\ Y_i(s_0) & Y_i(s_1) & \cdots & Y_i(s_{m-1}) \end{bmatrix}^{\mathrm{T}}, \tag{28}$$

$$Z^{(l)} = \begin{bmatrix} Z_l(s_0)Z_1(s_0) & Z_l(s_1)Z_1(s_1) & \cdots & Z_l(s_{m-1})Z_1(s_{m-1}) \\ Z_l(s_0)Z_2(s_0) & Z_l(s_1)Z_2(s_1) & \cdots & Z_l(s_{m-1})Z_2(s_{m-1}) \\ \cdots & \cdots & \cdots & \cdots \\ Z_l(s_0)Z_H(s_0) & Z_l(s_1)Z_H(s_1) & \cdots & Z_l(s_{m-1})Z_H(s_{m-1}) \\ Z_l(s_0) & Z_l(s_1) & \cdots & Z_l(s_{m-1}) \end{bmatrix}^{\mathrm{T}}. \tag{29}$$

Consequently, (18), (19) and (20) can be transformed into the following linear regression model:

$$U_i = X^{(i)}A_i + \varepsilon_i, \quad i = 1, 2, \ldots, N, \tag{30}$$

$$V_l = Y^{(l)}C_l + \varepsilon'_l, \quad l = 1, 2, \ldots, M, \tag{31}$$

$$W_p = Z^{(p)}G_p + \varepsilon''_p, \quad p = 1, 2, \ldots, H, \tag{32}$$

where $\varepsilon_i = (\varepsilon_{i0}, \varepsilon_{i1}, \ldots, \varepsilon_{i,m-1})^{\mathrm{T}}$, $\varepsilon'_l = (\varepsilon'_{l0}, \varepsilon'_{l1}, \ldots, \varepsilon'_{l,m-1})^{\mathrm{T}}$ and $\varepsilon''_p = (\varepsilon''_{p0}, \varepsilon''_{p1}, \ldots, \varepsilon''_{p,m-1})^{\mathrm{T}}$are the random effects. Here, each $\varepsilon_{ik},\ k = 0, 1, \ldots, m-1$ is the random disturbance with a mean of zero and $Cov(\varepsilon_i) = \sigma_i^2 I_m$, similarly, each $\varepsilon'_{lk},\ k = 0, 1, \ldots, m-1$ and is the random disturbance with a mean of zero and $Cov(\varepsilon'_l) = \sigma'^2_l I_m$, each $\varepsilon''_{pk},\ k = 0, 1, \ldots, m-1$ is the random disturbance with a mean of zero and $Cov(\varepsilon''_p) = \sigma''^2_p I_m$,

Now, we use an adapted Bayesian Lasso method to infer the posterior distribution over the coefficients in each A_i. The method of inferring the posterior distribution over the coefficients in each C_l and G_p is similar, so we will only describe in detail the process of inferring the posterior distribution over the coefficients in each A_i. First, the above assumptions imply that the data likelihood is

$$\ell(A_i, \sigma_i^2 | U_i, X^{(i)}) = \prod_{t=0}^{m-1} \phi(U_{it}; X_t^{(i)} A_i, \sigma_i^2), \tag{33}$$

where $t = 0, 1, \ldots, m-1$, $X_t^{(i)}$ is the $t+1$-th row of $X^{(i)}$ and $\phi(U_{it}; X_t^{(i)} A_i, \sigma_i^2)$ is the Gaussian probability density with mean $X_t^{(i)} A_i$ and variance σ_i^2 evaluated at U_{it}.

Then we assume that these prior distributions:

- $A_i | \sigma_i^2, \lambda_i$ has a Laplace distribution with a mean of 0 and a scale of $\frac{\sigma_i^2}{\lambda_i}$, where λ_i is the shrinkage parameter, which is set to 1. The coefficients are conditionally independent.
- $\sigma_i^2 \sim IG(A, B)$, where A and B are the shape and scale, respectively, of an inverse gamma distribution.

Using Bayes' rule, we formulate the joint posterior distribution of A_i and σ_i^2 as follows:

$$\pi(A_i, \sigma_i^2 | U_i, X^{(i)}) \propto \pi(A_i | \sigma_i^2, \lambda_i) \cdot \pi(\sigma_i^2) \cdot \ell(A_i, \sigma_i^2 | U_i, X^{(i)}). \tag{34}$$

Perform Bayesian lasso regression by passing the prior model and data to estimate, that is, by estimating the posterior distribution of A_i and σ_i^2 (C_l and σ'^2_l, G_p and σ''^2_p). Then we apply Markov chain Monte Carlo (MCMC) algorithm to sample from the posterior. A directed edge from AS event j (RBP l) to AS event i could be determined to be presented if the 95% credible interval (CI) of the parameter estimates of a_{ij} (b_{il}) does not contain zero, otherwise absent. Similarly, a directed edge from RBP k (TF p) to RBP l could be determined to be presented if the 95% credible interval (CI) of the parameter estimates of c_{lk} (e_{lp}) does not contain zero, otherwise absent. A directed edge from TF q to TF p could be determined to be presented if the 95% credible interval (CI) of the parameter estimates of g_{pq} does not contain zero, otherwise absent.
