# Supplementary material for: CTAS: a network control theory-based approach to identify key regulatory TFs of AS events during epithelial–mesenchymal transition
Source: Brief Bioinform. 2026 Feb 10;27(1):bbag042. doi: 10.1093/bib/bbag042 (PMC12888823; doi:10.1093/bib/bbag042)
Supplement: S5-Detailed_algorithm_bbag042 [file s5-detailed_algorithm_bbag042.pdf]

THE CTAS ALGORITHM FOR IDENTIFYING KEY TFS IN EMT-ASSOCIATED NETWORKS

Step 1. Identifying the target-controllable subsystem of each AS node

For a general bipartite graph the maximum matching can be obtained efficiently by the Hopcroft–Karp algorithm, whose running time is $\mathcal{O}\big(\sqrt{|V|}\,|E|\big)$ [3]. Consequently the worst-case complexity of our greedy expansion is $\mathcal{O}\big(r\sqrt{|V|}\,|E|\big)$, where r denotes the number of iterations, $|V|$ the number of vertices and $|E|$ the number of directed edges in $G(V,E)$.

Definition(Target–control configuration). Let $M = \bigcup_{k=0}^{r} m_k$ denote the union of all maximum matchings produced during the iterative expansion. The sub-graph $CF(V,M) = (V,M)$ is called the *target–control configuration*; it aggregates every directed link that participates in at least one matching layer.

We now state the key theoretical result.

Theorem (Target-controllable subsystem identification). For every target node $a \in \mathcal{A}$ the entire up-stream control set F_a can drive a in the full network $G(V,E)$.

Sketch of proof. Within $CF(V,M)$ each F_a forms a vertex-disjoint directed path ending at a, hence the subsystem lacks inaccessible vertices and dilations; structural controllability follows from Lin's theorem. Adding extra edges cannot destroy controllability [1]; therefore F_a controls a in $G(V,E)$. □

With the theorem we derive, for every TF $t \in \mathcal{T}$, the target-controllable subsystem $\mathrm{TCS}_t = \{\, a \in \mathcal{A} \mid t \in F_a \,\}$. If for two targets a, b the sets F_a and F_b overlap, both AS events are added to the corresponding subsystem. Note that Gao *et al.* [2] also iterated bipartite graphs, but with the aim of directly returning driver nodes. Our strategy differs: the iterative matchings are used solely to expose all target-controllable subspaces before any driver selection is carried out. Figure 2 in [2] illustrates that distinct matchings may lead to different driver-set sizes; replacing one link can never increase the minimum cardinality.

Step 2. Obtaining the TF driver set with minimal size via integer linear programming

Once the family of subsystems $\{\mathrm{TCS}_t\}_{t\in\mathcal{T}}$ is available, the task reduces to a minimum set-cover problem. Introduce binary variables x_t for each $t \in \mathcal{T}$; $x_t = 1$ indicates TF t is selected

as a driver. The problem is formulated as

$$\begin{aligned} \text{minimise} \ & \sum_{t \in \mathcal{T}} x_t \\ \text{subject to} \ & \sum_{t \in \mathrm{TCS}_a} x_t \ \geq 1, \quad \forall a \in \mathcal{A}, \\ & x_t \in \{0,1\}, \quad \forall t \in \mathcal{T}. \end{aligned}$$

Although the ILP is NP-hard, branch-and-bound solvers deliver optimal solutions for graphs up to several tens of thousands of variables within practical time [1].

Step 3. Refinement via Markov-chain sampling

Different target–control configurations may yield different objective values. To reduce sensitivity to an arbitrary matching sequence we perform a Markov-chain Monte-Carlo search. Starting from the initial configuration $\mathcal{M}^{(0)} = \bigcup_k m_k$, each iteration randomly chooses one layer m_k, flips an augmenting path to obtain a new maximum matching m'_k, and forms $\mathcal{M}^{\text{new}} = \big(\mathcal{M}^{(0)} \setminus m_k\big) \cup m'_k$.

For the updated configuration the ILP is re-solved; whenever a smaller driver set is found we update the incumbent solution $\mathcal{K}^*$. The process stops after a pre-defined number of successful non-worsening moves or when a time budget is exhausted.

Figure 1 presents a step-by-step illustration of the CTCA procedure on a small example network. The target node set is defined as $O = \{v_3, v_4, v_8\}$, shown as green circles, and the constrained control node set is $U = \{v_1, v_2\}$, shown as pentagons.

First, a layered connection–dynamic graph is constructed by iteratively building bipartite graphs. In each iteration, the left-side nodes from the previous matching are used as the right-side nodes of the current bipartite graph, and the Hopcroft–Karp algorithm is applied to find a maximum matching. After three iterations, the algorithm produces a complete layered graph. The union of matched edges across all layers forms a set of paths, shown in red, that connect constrained control nodes to target nodes.

According to the matching paths, we obtain three control paths ending at the target nodes:

$$\begin{aligned} F_{v_3} &= \{v_3, v_1\}, \\ F_{v_4} &= \{v_4, v_3, v_2\}, \\ F_{v_8} &= \{v_8, v_1, v_3, v_2\}. \end{aligned}$$

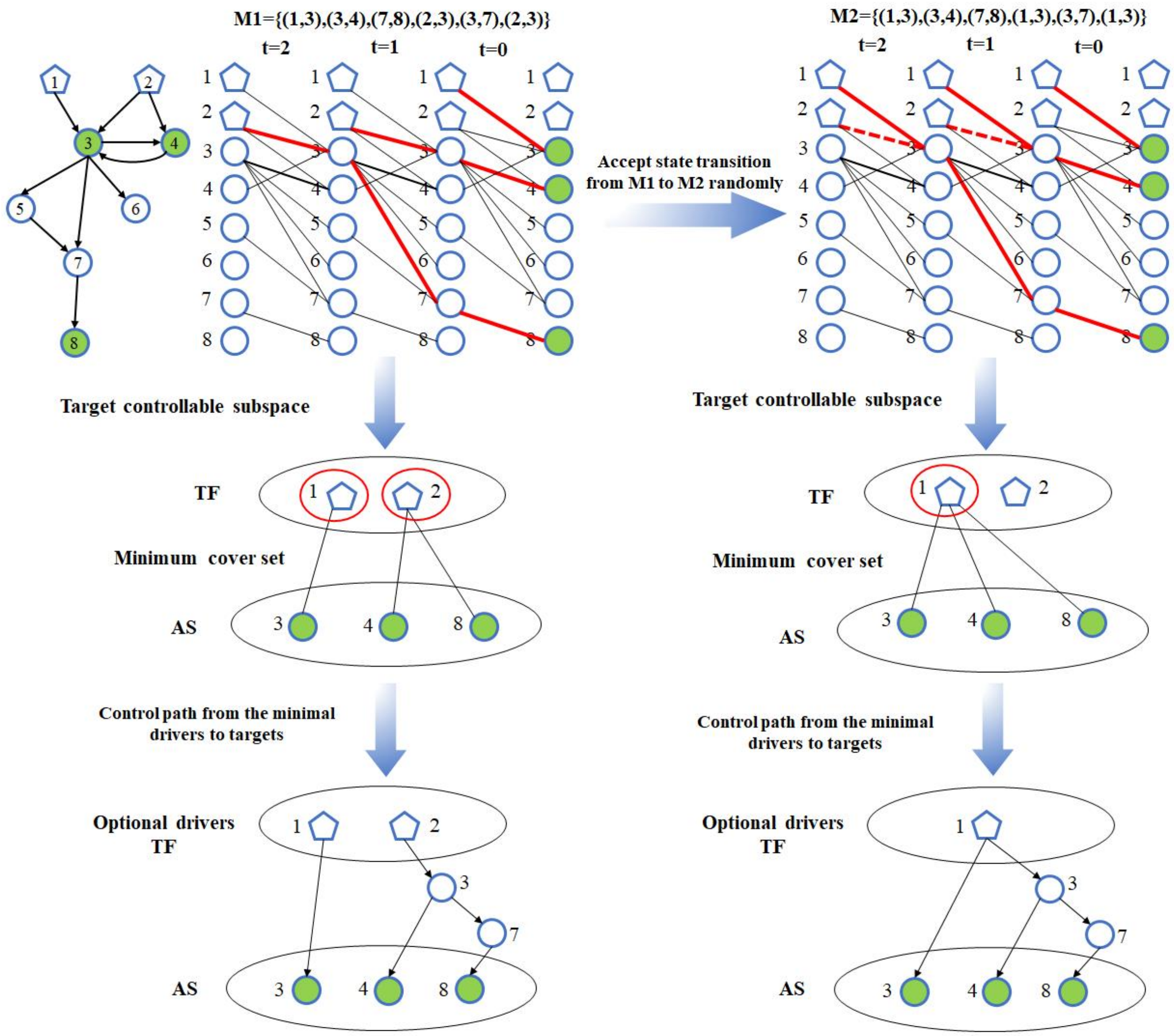

Fig. 1: The procedure of algorithm for CTAS.

These are the upstream control sets, consisting of all nodes along the paths controlling each target.

From these, we can derive the target-controllable subsystems of the constrained nodes. For instance, node v_2 controls v_4 and v_8 through its reachable paths, so

$$\mathrm{TCS}_{v_2} = \{v_4, v_8\}, \quad \mathrm{TCS}_{v_1} = \{v_3\}.$$

Finally, based on all TCS_t, we solve the minimum set cover problem to identify the minimal set of constrained drivers. In this example, the optimal driver set is $\{v_1, v_2\}$, which together

control all targets $\{v_3, v_4, v_8\}$. At the same time, the algorithm yields explicit control paths from each driver to its associated targets.

Once the target-controllable subsystems are computed, the minimum set cover approximation ensures the smallest set of constrained control nodes that collectively control the specified targets.

REFERENCES

[1] Guo W.-F. *et al*. Constrained target controllability of complex networks. *Journal of Statistical Mechanics: Theory and Experiment* **2017**(6) 063402.

[2] Gao J., Liu Y.-Y., D'Souza R.M., Barabási A.-L. Target control of complex networks. *Nature Communications* **5** (2014) 5415.

[3] Hopcroft J.E., Karp R.M. An $n^{5/2}$ algorithm for maximum matchings in bipartite graphs. *SIAM Journal on Computing* **2** (1973) 225–231.
