# Supplementary material for: CTAS: a network control theory-based approach to identify key regulatory TFs of AS events during epithelial–mesenchymal transition
Source: Brief Bioinform. 2026 Feb 10;27(1):bbag042. doi: 10.1093/bib/bbag042 (PMC12888823; doi:10.1093/bib/bbag042)
Supplement: S6-Robustness_analysis_bbag042 [file s6-robustness_analysis_bbag042.pdf]

MATHEMATICAL PROOF

Considering the technical variability or measurement error in the transcriptomic data [1], [2], [3], it is important to examine the robustness of the method with respect to the perturbation in pseudo-time progression. In this part, the robustness of model to the measured variables in the data is illustrated by mathematical proof.

Theorem 1. *Assume that there are two trajectories of pseudo-time progression $s(r)$ and $\tilde{s}(r)$ with the same root $r \in I = [0,1]$. Define $\|\tilde{s}-s\|_{L^2} = (\int_I |\tilde{s}-s|^2 dr)^{1/2}$. If $(X_i(s), Y_l(s), Z_p(s), a_{ij}, b_{il}, c_{lk}, e_{lp}, g_{pq})$ and $(X_i(\tilde{s}), Y_l(\tilde{s}), Z_p(\tilde{s}), \tilde{a}_{ij}, \tilde{b}_{il}, \tilde{c}_{lk}, \tilde{e}_{lp}, \tilde{g}_{pq})$ both satisfy the equations of progression-dependent dynamic model, i.e.,*

$$\frac{\mathrm{d}X_i(s)}{\mathrm{d}s} = \sum_{j\neq i} a_{ij}X_i(s)\cdot X_j(s) + \sum_{l=1}^{M} b_{il}X_i(s)\cdot Y_l(s) - d_iX_i(s), \quad i = 1,2,\ldots,N, \tag{1}$$

$$\frac{\mathrm{d}Y_l(s)}{\mathrm{d}s} = \sum_{k\neq l} c_{lk}Y_l(s)\cdot Y_k(s) + \sum_{p=1}^{H} e_{lp}Y_l(s)\cdot Z_p(s) - d_l'Y_l(s), \quad l = 1,2,\ldots,M, \tag{2}$$

$$\frac{\mathrm{d}Z_p(s)}{\mathrm{d}s} = \sum_{p\neq q} g_{pq}Z_p(s)\cdot Z_q(s) - d_p''Z_p(s), \quad p = 1,2,\ldots,H, \tag{3}$$

$$\frac{\mathrm{d}X_i(\tilde{s})}{\mathrm{d}\tilde{s}} = \sum_{j\neq i} \tilde{a}_{ij}X_i(\tilde{s})\cdot X_j(\tilde{s}) + \sum_{l=1}^{M} \tilde{b}_{il}X_i(\tilde{s})\cdot Y_l(\tilde{s}) - \tilde{d}_iX_i(\tilde{s}), \quad i = 1,2,\ldots,N, \tag{4}$$

$$\frac{\mathrm{d}Y_l(\tilde{s})}{\mathrm{d}\tilde{s}} = \sum_{k\neq l} \tilde{c}_{lk}Y_l(\tilde{s})\cdot Y_k(\tilde{s}) + \sum_{p=1}^{H} \tilde{e}_{lp}Y_l(\tilde{s})\cdot Z_p(\tilde{s}) - \tilde{d}_l'Y_l(\tilde{s}), \quad l = 1,2,\ldots,M, \tag{5}$$

$$\frac{\mathrm{d}Z_p(\tilde{s})}{\mathrm{d}s} = \sum_{p\neq q} g_{pq}Z_p(\tilde{s})\cdot Z_q(\tilde{s}) - \tilde{d}_p''Z_p(\tilde{s}), \quad p = 1,2,\ldots,H, \tag{6}$$

then we have

$$\lim_{\|\tilde{s}-s\|_{L^2}\to 0} \left(\sum_{j=1}^{N} (\tilde{a}_{ij} - a_{ij})^2 + \sum_{l=1}^{M} (\tilde{b}_{il} - b_{il})^2 \right) = 0, \tag{7}$$

$$\lim_{\|\tilde{s}-s\|_{L^2}\to 0} \left(\sum_{k=1}^{M} (\tilde{c}_{lk} - c_{lk})^2 + \sum_{p=1}^{H} (\tilde{e}_{lp} - e_{lp})^2 \right) = 0, \tag{8}$$

and

$$\lim_{\|\tilde{s}-s\|_{L^2}\to 0} \left(\sum_{q=1}^{H} (\tilde{g}_{pq} - g_{pq})^2 \right) = 0. \tag{9}$$

Proof: We first transform the (1) into the following form,

$$\frac{\mathrm{d}(\log X_i(s))}{\mathrm{d}s} = \sum_{j\neq i} a_{ij}X_j(s) + \sum_{l=1}^{M} b_{il}Y_l(s) - d_i, \tag{10}$$

Denoting $a_{ii} = 0$ and integrating the above equation leads to

$$\log X_i(s) = \sum_{j=1}^{N} a_{ij}\int_0^s X_j(\tau)d\tau + \sum_{l=1}^{M} b_{il}\int_0^s Y_l(\tau)d\tau - d_i s + \log X_i(0). \tag{11}$$

Denote $U_i(s) = \log X_i(s),\ V_j(s) = \int_0^s X_j(\tau)d\tau$ and $W_l(s) = \int_0^s Y_l(\tau)d\tau$. The above equation can be written as

$$\begin{aligned} U_i(s) =& \sum_{j=1}^{N} a_{ij}V_j(s) + \sum_{l=1}^{M} b_{il}W_l(s) - d_i s + U_i(0) \\ =& \sum_{j=1}^{N}(a_{ij} - \tilde{a}_{ij})V_j(s) + \sum_{l=1}^{M}(b_{il} - \tilde{b}_{il})W_l(s) + \sum_{j=1}^{N}\tilde{a}_{ij}V_j(s) + \sum_{l=1}^{M}\tilde{b}_{il}W_l(s) - d_i s + U_i(0) \\ =& \sum_{j=1}^{N}(a_{ij} - \tilde{a}_{ij})V_j(s) + \sum_{l=1}^{M}(b_{il} - \tilde{b}_{il})W_l(s) + \sum_{j=1}^{N}\tilde{a}_{ij}\left(V_j(s) - V_j(\tilde{s})\right) + \\ & \sum_{l=1}^{M}\tilde{b}_{il}\left(W_l(s) - W_l(\tilde{s})\right) + \left[\sum_{j=1}^{N}\tilde{a}_{ij}V_j(\tilde{s}) + \sum_{l=1}^{M}\tilde{b}_{il}W_l(\tilde{s}) - \tilde{d}_i\tilde{s} + U_i(0)\right] + \tilde{d}_i\tilde{s} - d_i s \\ =& \sum_{j=1}^{N}(a_{ij} - \tilde{a}_{ij})V_j(s) + \sum_{l=1}^{M}(b_{il} - \tilde{b}_{il})W_l(s) + U_i(\tilde{s}) + \sum_{j=1}^{N}\tilde{a}_{ij}\left(V_j(s) - V_j(\tilde{s})\right) + \\ & \sum_{l=1}^{M}\tilde{b}_{il}\left(W_l(s) - W_l(\tilde{s})\right) - \tilde{d}_i(s - \tilde{s}) - (d_i - \tilde{d}_i)s. \end{aligned} \tag{12}$$

Therefore, we have

$$\begin{aligned} & \sum_{j=1}^{N}(a_{ij} - \tilde{a}_{ij})V_j(s) + \sum_{l=1}^{M}(b_{il} - \tilde{b}_{il})W_l(s) - (d_i - \tilde{d}_i)s \\ =& U_i(s) - U_i(\tilde{s}) - \sum_{j=1}^{N}\tilde{a}_{ij}\left(V_j(s) - V_j(\tilde{s})\right) - \sum_{l=1}^{M}\tilde{b}_{il}\left(W_l(s) - W_l(\tilde{s})\right) + \tilde{d}_i(s - \tilde{s}). \end{aligned} \tag{13}$$

For $i = 1, 2, \ldots, N$ and $j = 1, \ldots, N + M$, define

$$w_{ij} = \begin{cases} a_{ij} - \tilde{a}_{ij}, \quad i \neq j,\ j = 1, \ldots, N \\ -(d_i - \tilde{d}_i), \quad i = j,\ j = 1, \ldots, N \\ b_{i,j-N} - \tilde{b}_{i,j-N}, \quad j = N+1, \ldots, N+M \end{cases}$$

and redefine $U_j(s) = \begin{cases} \displaystyle\int_0^s X_j(\tau)d\tau, \quad i \neq j,\ j = 1, \ldots, N \\ s, \quad i = j,\ j = 1, \ldots, N \\ \displaystyle\int_0^s Y_{j-N}(\tau)d\tau, \quad j = N+1, \ldots, N+M \end{cases}$

Denote $R_i = (r_{i1}, r_{i2}, \ldots, r_{i,N+M})^{\mathrm{T}}$, $V = (V_1(s), V_2(s), \ldots, V_{N+M}(s))$ and $Ti = U_i(s) - U_i(\tilde{s}) - \sum_{j=1}^{N} \tilde{a}_{ij} (V_j(s) - V_j(\tilde{s})) - \sum_{l=1}^{M} \tilde{b}_{il} (W_l(s) - W_l(\tilde{s})) + \tilde{d}_i(s-\tilde{s})$. Then (13) can be written as

$$VR_i = T_i. \tag{14}$$

Take $m+1$ points on $s(\tau)$ and $\tilde{s}(\tau)$, respectively. Here, $s_i = s(\tau_i)$ and $\tilde{s}_i = \tilde{s}(\tau_i)$, where $\tau_i = \frac{i}{m}$, $i = 0, 1, \ldots, m$. Let $\mathbb{T}_i = (T_i(r_0), T_i(r_1), \ldots, T_i(r_{m-1}))^{\mathrm{T}}$ and

$$\mathbb{U} = \begin{bmatrix} U_1(s_0) & U_2(s_0) & \cdots & U_{N+M}(s_0) \\ U_1(s_1) & U_2(s_1) & \cdots & U_{N+M}(s_1) \\ \vdots & \vdots & \ddots & \vdots \\ U_1(s_{m-1}) & U_2(s_{m-1}) & \cdots & U_{N+M}(s_{m-1}) \end{bmatrix}_{m\times(N+M)} . \tag{15}$$

Then we have

$$\mathbb{U}R_i = \mathbb{T}_i. \tag{16}$$

Therefore, we have

$$\hat{R}_i = \arg\min_{R_i} \|\mathbb{T}_i - \mathbb{U}R_i\|^2 = \arg\min_{R_i} (\mathbb{T}_i - \mathbb{U}R_i^{\mathrm{T}}(\mathbb{T}_i - \mathbb{U}R_i). \tag{17}$$

By matrix derivation, we get

(a) When $rank(\mathbb{U}) = N+M$,

$$\hat{R}_i = (\mathbb{U}^{\mathrm{T}}\mathbb{U})^{-1}\mathbb{U}^{\mathrm{T}}\mathbb{T}_i. \tag{18}$$

(b) When $rank(\mathbb{U}) \neq N+M$, there exists $\lambda > 0$ such that

$$\hat{R}_i = (\mathbb{U}^{\mathrm{T}}\mathbb{U} + \lambda I_{N+M})^{-1}\mathbb{U}^{\mathrm{T}}\mathbb{T}_i. \tag{19}$$

From the definition of R_i, (18) and (19), we know that

$$\sum_{j=1}^{N} (a_{ij} - \tilde{a}_{ij})^2 + \sum_{l=1}^{M} (b_{il} - \tilde{b}_{il})^2 + (d_i - \tilde{d}_i)^2 \leq \alpha_1 \cdot \|\mathbb{T}_i\|_{L^2}^2, \tag{20}$$

where α_1 is a positive constant. According to the definition of $\mathbb{T}_i$, we have

$$\|\mathbb{T}_i\|_{L^2} \leq \alpha_2 \|T_i\|_{L^2}, \tag{21}$$

where α_2 is a positive constant and $\|T_i\|_{L^2} \leq \|U_i(s)-U_i(\tilde{s})\|_{L^2} + \|\sum_{j=1}^{N}\tilde{a}_{ij}(V_j(s)-V_j(\tilde{s}))\|_{L^2} + \|\sum_{l=1}^{M}\tilde{b}_{il}(W_l(s)-W_l(\tilde{s}))\|_{L^2} + \tilde{d}_i\|s-\tilde{s}\|_{L^2}$. Now we only need to prove that the first three terms in the right hand of the above inequality tends to 0 as $\|s-\tilde{s}\| \to 0$.

(i) We first prove $\lim_{\|s-\tilde{s}\|\to 0}\|U_i(s)-U_i(\tilde{s})\|_{L^2} = 0$. Since $U_i(s) = \log X_i(s)$, we only need to prove $\lim_{\|s-\tilde{s}\|\to 0}\|X_i(s)-X_i(\tilde{s})\|_{L^2} = 0$, which is valid according to the continuity of $X_i(s)$ with respect to s.

(ii) Then we prove $\lim_{\|s-\tilde{s}\|\to 0}\|\sum_{j=1}^{N}\tilde{a}_{ij}(V_j(s)-V_j(\tilde{s}))\|_{L^2} = 0$.

$$\begin{aligned}
&\sum_{j=1}^{N}\tilde{a}_{ij}(V_j(s)-V_j(\tilde{s})) \\
&= -\sum_{j=1}^{N}\tilde{a}_{ij}\left(\int_0^{\tilde{s}}X_j(\tau)d\tau - \int_0^{s}X_j(\tau)d\tau\right) \\
&= -\sum_{j=1}^{N}\tilde{a}_{ij}\left[\lim_{m\to\infty}\sum_{l=0}^{m-1}\left(\int_{\tilde{s}_l}^{\tilde{s}_{l+1}}X_j(\tau)d\tau - \int_{s_l}^{s_{l+1}}X_j(\tau)d\tau\right)\right].
\end{aligned} \tag{22}$$

For any m, since $\|s-\tilde{s}\| \to 0$, we have $\tilde{s}_l \to s_l,\ l = 0, 1, \ldots, m$ and thus

$$\begin{aligned}
&\lim_{\|s-\tilde{s}\|\to 0}\sum_{l=0}^{m-1}\left(\int_{\tilde{s}_l}^{\tilde{s}_{l+1}}X_j(\tau)d\tau - \int_{s_l}^{s_{l+1}}X_j(\tau)d\tau\right) \\
=&\sum_{l=1}^{m-1}\lim_{\|s-\tilde{s}\|\to 0}\left(\int_{\tilde{s}_l}^{\tilde{s}_{l+1}}X_j(\tau)d\tau - \int_{s_l}^{s_{l+1}}X_j(\tau)d\tau\right).
\end{aligned} \tag{23}$$

That is, the above limit is uniformly convergent with respect to m. Therefore

$$\begin{aligned}
&\lim_{\|s-\tilde{s}\|\to 0}\left(\int_0^{\tilde{s}}X_j(\tau)d\tau - \int_0^{s}X_j(\tau)d\tau\right) \\
=&\lim_{\|s-\tilde{s}\|\to 0}\lim_{m\to\infty}\sum_{l=0}^{m-1}\left(\int_{\tilde{s}_l}^{\tilde{s}_{l+1}}X_j(\tau)d\tau - \int_{s_l}^{s_{l+1}}X_j(\tau)d\tau\right) \\
=&\lim_{m\to\infty}\sum_{l=0}^{m-1}\lim_{\|s-\tilde{s}\|\to 0}\left(\int_{\tilde{s}_l}^{\tilde{s}_{l+1}}X_j(\tau)d\tau - \int_{s_l}^{s_{l+1}}X_j(\tau)d\tau\right) \\
=&0.
\end{aligned} \tag{24}$$

From the above (22), (23) and (24), we can obtain that

$$\lim_{\|s-\tilde{s}\|\to 0}\|\sum_{j=1}^{N}\tilde{a}_{ij}(V_j(s)-V_j(\tilde{s}))\|_{L^2} = 0.$$

(iii) Similarly, we have $\lim_{\|s-\tilde{s}\|\to 0}\|\sum_{l=1}^{M}\tilde{b}_{il}(W_l(s)-W_l(\tilde{s}))\|_{L^2} = 0$.

Therefore, (7) holds. Similarly, we can prove that (8) and (9) holds. ■

Based on the spectral graph theory [4], [5], the above manifold distance is noise-resistant, so the variation in the progression trajectory, i.e., $\tilde{s} - s$, should be small considering the moderate perturbations. As a result, Theorem 1 means that the corresponding estimates of $[a_{ij}]_{N\times N},$ $[b_{il}]_{N\times M}, [c_{lk}]_{M\times M}, [e_{lp}]_{M\times H}$ and $[g_{pq}]_{H\times H}$ should vary minimally. Therefore, the above theorem theoretically ensures the consistency and robustness of the regulatory coefficient estimation. Moreover, the Bayesian Lasso method used in this paper can further guarantee a robust implementation of network inference.

A corollary of the above theorem is that the mappings $s \mapsto [a_{ij}(s)]_{N\times N},$ $s \mapsto [b_{il}(s)]_{N\times M}, s \mapsto [c_{lk}(s)]_{M\times M}, s \mapsto [e_{lp}(s)]_{M\times H}$ and $s \mapsto [g_{pq}(s)]_{H\times H}$ are continuous under some appropriate metric. More specifically, for two trajectories s and $\tilde{s}$, if the difference between the two inferred regulatory coefficients $[a_{ij}(s)]_{N\times N}, [b_{il}(s)]_{N\times M}, [c_{lk}(s)]_{M\times M}, [e_{lp}(s)]_{M\times H}, [g_(s)pq]_{H\times H}$ and $[a_{ij}(\tilde{s})]_{N\times N}, [b_{il}(\tilde{s})]_{N\times M}, [c_{lk}(\tilde{s})]_{M\times M}, [e_{lp}(\tilde{s})]_{M\times H}, [g_{pq}(\tilde{s})]_{H\times H}$ is notably greater than 0, then the difference between s and $\tilde{s}$ should not be arbitrarily small. This means that if the inferred regulatory networks for two progressions are largely different, then the two progressions should have distinct trajectories, resulting in distinct different clinical outcomes. Therefore, Theorem 1 also shows that our proposed model may be used to predict or control cancer progression.

REFERENCES

[1] Ji,H., and Davis,R.W. (2006). Data quality in genomics and microarrays, *Nature Biotechnology*, **24(9)**, 1112–1113.

[2] Nie,L. *et al.* (2007). Integrative analysis of transcriptomic and proteomic data: challenges, solutions and applications, *Critical Reviews in Biotechnology*, **27(2)**, 63–75.

[3] Su,Z. *et al.* (2014). A comprehensive assessment of RNA-seq accuracy, reproducibility and information content by the Sequencing Quality Control Consortium, *Nature Biotechnology*, **32(9)**, 903–914.

[4] Chung,F.R., and Graham,F.C. (1997). *Spectral graph theory*, American Mathematical Soc.

[5] Coifman,R.R., and Lafon,S. (2006). Diffusion maps, *Applied and Computational Harmonic Analysis*, **21(1)**, 5–30.
