# Supplementary material for: CTAS: a network control theory-based approach to identify key regulatory TFs of AS events during epithelial–mesenchymal transition
Source: Brief Bioinform. 2026 Feb 10;27(1):bbag042. doi: 10.1093/bib/bbag042 (PMC12888823; doi:10.1093/bib/bbag042)
Supplement: S7-Testing_method_with_a_synthetic_dataset_bbag042 [file s7-testing_method_with_a_synthetic_dataset_bbag042.pdf]

Testing the model with a synthetic dataset

To assess the performance of the CTAS model in recovering regulatory structure and pseudotemporal dynamics, we constructed a synthetic dataset that mimics the expression of transcription factors (TFs), RNA-binding proteins (RBPs), and alternative splicing (AS) events during epithelial–mesenchymal transition (EMT). The simulation involved 2 TFs, 3 RBPs, and 5 AS events across 100 virtual samples.

We formulated the dynamic regulatory system using a three-layer ordinary differential equation (ODE) model:

$$\frac{\mathrm{d}X_i(s)}{\mathrm{d}s} = \sum_{j\neq i} a_{ij}X_i(s)\cdot X_j(s) + \sum_{l=1}^{M} b_{il}X_i(s)\cdot Y_l(s) - d_iX_i(s), \quad i = 1,2,\ldots,N, \tag{1}$$

$$\frac{\mathrm{d}Y_l(s)}{\mathrm{d}s} = \sum_{k\neq l} c_{lk}Y_l(s)\cdot Y_k(s) + \sum_{p=1}^{H} e_{lp}Y_l(s)\cdot Z_p(s) - d_l'Y_l(s), \quad l = 1,2,\ldots,M, \tag{2}$$

$$\frac{\mathrm{d}Z_p(s)}{\mathrm{d}s} = \sum_{p\neq q} g_{pq}Z_p(s)\cdot Z_q(s) - d_p''Z_p(s), \quad p = 1,2,\ldots,H, \tag{3}$$

Here, $X_i(s)$, $Y_l(s)$, and $Z_p(s)$ represent the expression levels of AS, RBP, and TF nodes, respectively, at pseudotime s. The regulatory coefficients were defined as follows:

$$(a_{ij})_{5\times5} = \begin{bmatrix} 0 & -2 & 0 & 0 & 2 \\ 0 & 0 & -1 & 0 & 0 \\ -1 & 0 & 0 & 1 & 0 \\ 4 & 0 & -3 & 0 & 0 \\ 0 & 0 & -2 & 1 & 0 \end{bmatrix}, \quad (b_{il})_{5\times3} = \begin{bmatrix} -1 & 0 & 0 \\ 0 & 1 & 0 \\ 0 & 0 & 0 \\ 0 & 1 & 1 \\ 2 & 0 & 0 \end{bmatrix},$$

$$(c_{lk})_{3\times3} = \begin{bmatrix} 0 & -2 & 1 \\ 1 & 0 & -2 \\ 0 & -1 & 0 \end{bmatrix}, \quad (e_{lp})_{3\times2} = \begin{bmatrix} -1 & 0 \\ 0 & 1 \\ 2 & 0 \end{bmatrix}, \quad (g_{pq})_{2\times2} = \begin{bmatrix} 0 & -2 \\ 1 & 0 \end{bmatrix}.$$

$$d_i = (-1,\ -1,\ -0.5,\ -1,\ -0.5)^{\mathrm{T}}, \quad d_l' = (-1,\ -1,\ -0.5)^{\mathrm{T}}, \quad d_p'' = (-1,\ -0.5)^{\mathrm{T}}.$$

All nodes were initialized to 1. The system was numerically integrated using an ODE solver to simulate the dynamic expression profiles along pseudotime $s \in [0, 10]$. To mimic the structure of

cross-sectional data, samples were drawn from two distinct sub-intervals: $S_1 = [0, 4.5]$ (epithelial-like state) and $S_2 = [5.5, 10]$ (mesenchymal-like state). A total of 50 samples were uniformly sampled from each interval, and sample identifiers were randomly permuted while retaining their S1/S2 labels.

a) Evaluation metrics: We assessed the quality of the inferred pseudotime by comparing the predicted order to the ground-truth sample order using Spearman's rank correlation coefficient ρ and root mean square error (RMSE).

To evaluate the reconstruction of regulatory relationships, Bayesian Lasso regression was applied to infer the edge weights. A confidence score for each predicted edge was computed as:

$$S_{ij} = 1 - \inf \{\alpha \in (0, 1) \mid 0 \notin \mathrm{CI}_\alpha([A_i]_{ij})\}, \tag{4}$$

where CI_α denotes the α-level Bayesian credible interval for coefficient $[A_i]_{ij}$.

Using different thresholds on S_{ij}, we calculated the true positive rate (TPR) and false positive rate (FPR) to plot the receiver operating characteristic (ROC) curve:

$$\mathrm{TPR} = \frac{\text{TP}}{\text{TP} + \text{FN}}, \quad \mathrm{FPR} = \frac{\text{FP}}{\text{TN} + \text{FP}}. \tag{5}$$

The area under the ROC curve (AUC) was used to quantify the inference accuracy of the network. In simulation experiments, CTAS achieved a Spearman correlation of 0.99946 and AUC = 0.94 under noise-free conditions, indicating high fidelity in pseudotime ordering and network reconstruction.
